# Application of palladium nanoparticles supported on ordered mesoporous oxides for C–N and C≡C coupling reactions in water†

**DOI:** 10.1039/d5ra02824h

**Published:** 2025-07-29

**Authors:** Nomathamsanqa Prudence Maqunga, Matumuene Joe Ndolomingo, Ndzondelelo Bingwa, Reinout Meijboom

**Affiliations:** a Department of Chemical Sciences, University of Johannesburg PO Box 524, Auckland Park Johannesburg 2006 South Africa rmeijboom@uj.ac.za +27 (0)11 559 2819 +27 (0)11 559 2367

## Abstract

This study sought to synthesize supported palladium nanocatalysts that are, in general, convenient to synthesize, suitable for mild conditions, recyclable, and stable in water. The sol–gel procedure was successfully extended to synthesize mesoporous metal oxides with well-dispersed palladium nanoparticles. The resulting catalysts were extensively characterized using techniques such as TEM, powder XRD, SEM-EDX, thermogravimetric analysis, and BET surface area measurements. The catalytic activity of the prepared heterogeneous palladium nanoparticles supported on mesoporous oxides was investigated in terms of C–N and C≡C coupling reactions, yielding products of alkynes and *N*-arylamines. Specifically, alkynes were effectively cross-coupled with various aryl iodides and aryl bromides, yielding diaryl alkynes with high efficiency and minimal catalyst loss. Similarly, the Buchwald–Hartwig amination reaction produced its desired products with high selectivity and yield.

## Abstract 2

This study sought to synthesize supported palladium nanocatalysts that are, in general, convenient to synthesize, suitable for mild conditions, recyclable, and stable in water.

## Graphical abstract

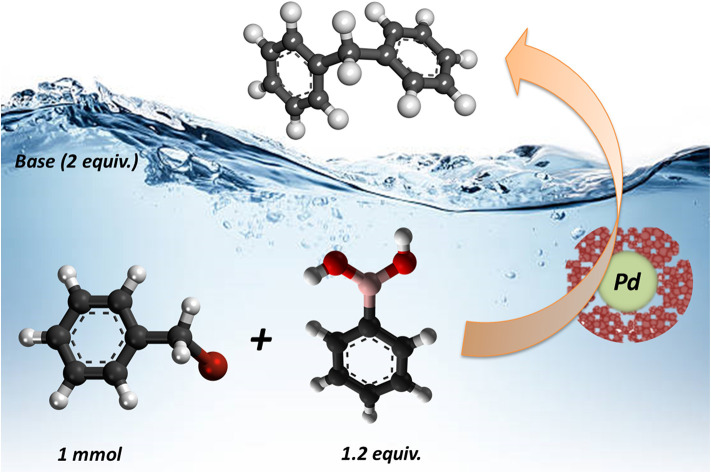

## 1. Introduction

Palladium-catalyzed cross-coupling has been a prominent approach for creating effective C–C and C-heteroatom bonds in synthesizing pharmaceuticals, fine chemicals, and novel materials in recent decades.^1–4^ Typically, cross-coupling reactions are carried out under homogeneous conditions, and rely on a catalyst, such as a soluble organic palladium complex, to occur efficiently.^5^ The complexing ligands, such as phosphine and amine derivatives, are, however, expensive and risk making the catalysts moisture-sensitive.^4,6^ Moreover, due to their high cost, low yield in separation, and low reusability, homogeneous transition metal catalysts remain a scientific challenge.^4,5^ While there are limitations, they can be overcome by the deposition of metal nanoparticles (MNPs) onto solid supports to fabricate what is commonly referred to as heterogeneous catalysts.^7^ Various forms of palladium-based heterogeneous catalysts have been developed for cross-coupling reactions, including supported, graphene-based and mixed metal oxide nanocatalysts.^1^ Among these, palladium supported nanocatalysts have gained extensive attention in both academia and industry because of their versatility in C–C and C-heteroatom cross-coupling reactions.^1,8,9^

Supported palladium nanocatalysts are transforming the landscape of modern catalysis and offer sustainable and efficient alternatives to traditional catalysts. Owing to their exceptional surface area-to-volume ratio, palladium nanoparticles exhibit remarkable catalytic activity and selectivity. Their high surface area-to-volume ratio facilitates their widespread use in cross-coupling reactions, thereby addressing critical challenges such as nanoparticle aggregation, palladium leaching, and environmental toxicity.^1^ This property makes them ideal for greener chemical processes. In most industrial applications, heterogeneous catalysts have proven to be the best alternative for homogeneous systems, and have been preferred over homogeneous catalysts.^7,10^

Recent advancements in palladium supported catalysis have underscored the significant role of innovative support materials in enhancing catalytic performance.^1^ A variety of heterogeneous solid-supported palladium catalysts, including silica, zeolite, mesoporous materials, and others, have been found to perform cross-coupling reactions.^5,10^ To maximize catalytic activity, metal nanoparticles are commonly deposited onto support materials, such as mesoporous metal oxides (MMOs).^11^ Mesoporous metal oxide materials contain organized pore structures that have dimensions comparable to metal nanoparticles in size.^12,13^ Their tunable structural properties such as, surface area, pore volume and pore sizes lead to a range of applications.

Using mesoporous metal oxides in heterogeneous catalysis offers two key advantages to nanoparticles (NPs): they prevent clumping and improve their thermal stability.^11,14^ Furthermore, metal oxides enhance their catalytic activity through a synergistic effect by providing surrounding acid/base and redox sites or desired metal/metal oxide interfaces.^15^

These developments have demonstrated that the rational design of MNPs/MMOs systems can lessen many of the challenges associated with traditional catalysts, offering unprecedented control over their stability, selectivity, and reusability. Designing and preparing efficient, stable, and cost-effective MNPs/MMOs has been a primary focus in many applications, particularly in the catalysis field.^16,17^ Herein, we attempted the synthesis of novel transition metal oxide nanoparticles and got more insights into their potential for application in organic synthesis. We specifically focused on their potential in Sonogashira and Buchwald–Hartwig coupling reactions, which are important in organic synthesis.

The Sonogashira cross-coupling reaction is used to connect the C–C bonds between aryl halides and aryl alkynes, resulting in the formation of C(sp^2^)–C(sp) bonds, which is an important process in organic synthesis.^18,19^ The synthesis of substituted alkynes has relied heavily on the results of these reactions. To enhance the reactions' effectiveness, copper salts have long been incorporated as co-catalysts in the Sonogashira process.^19^ This methodology has been extensively applied for the synthesis of natural products and biologically active drugs, as well as polymeric, non-linear optical and molecular electronic materials.^20,21^

The Buchwald–Hartwig reaction is a synthetic organic chemistry reaction that involves Pd-mediated cross-coupling of amines and aryl halides to produce C–N bonds.^2^ Cross-coupling processes can generate this C–N bond, which can be used to make a variety of molecules in the biological, pharmaceutical and material sciences.^4,5^ The Buchwald–Hartwig reaction offers a faster and simpler way to synthesize arylamines, replacing more time-consuming methods such as the Goldberg reaction and rigorous nucleophilic aromatic substitution, while greatly boosting (expanding) the collection of potential carbon–nitrogen bond formation.^2^

In the chemical and pharmaceutical industries, recycling catalysts is a task with significant economic and environmental benefits, especially when expensive or toxic heavy metal compounds are used.^22,23^ Due to its high cost and toxicity, removing palladium from organic compounds at the end of the reaction is a crucial goal.^22,23^ Developing ligand-free immobilized palladium catalysts is a difficult and significant challenge in terms of environmentally friendly organic synthesis.^24^ Therefore, the introduction of new catalyst systems and their applicability in the cross-coupling of aryl halides with amines is receiving special attention.^5^ The appeal of using water as a solvent, the absence of harmful chemicals, the ease of set-up and cost-effectiveness have all garnered interest in current research.^25^ This study details Sonogashira and Buchwald–Hartwig coupling reactions conducted in water using cost-effective in-house synthesized supported palladium-based catalysts. These catalysts feature palladium nanoparticles deposited on various mesoporous metal oxide materials, including cerium, cobalt, manganese, nickel, and titanium oxides. Traditionally, these types of cross-coupling reactions are performed in the presence of ligand complexes. However, this work attempted a fully heterogenized system in water.

## 2. Experimental

### 2.1 Materials

Cobalt(ii) nitrate hexahydrate (≥98.0%), nickel(ii) nitrate hexahydrate (≥99%), cerium(ii) nitrate hexahydrate (≥99.0%), titanium(iv) isoporoxide (97%), manganese(ii) nitrate tetrahydrate (≥97%), poly(ethylene glycol)-*block*-poly(propylene glycol)-*block*-poly(ethylene glycol) (Pluronic P-123) (99%), sodium borohydride (≥98%), urea (≥98%), aniline (95%), *O*-toluidine (98%), phenylacetylene (98%), chloroform-d (99.8%), 4-bromoacetophenone (98%), 4-bromobenzonitrile (99%), 4-bromoanisole (99%), 4-bromotoluene (≥99%), 4-bromobenzaldehyde (99%), 4-bromobenzene (≥99.5%), 4-bromoaniline (97%), 4-iodobenzene (99%), 2-iodoanisole (98%), benzyl bromide (98%), 1-iodo-2-nitrobenzene (97%), ethyl acetate (98%) and hydroxypropyl methylcellulose [HPMC, (98%)] were all purchased from Sigma Aldrich. Anhydrous magnesium sulphate [MgSO_4_, (≥99%)] and potassium carbonate [K_2_CO_3_, (≥99.5%)] were purchased from Merck Chemicals (PTY) Ltd. All experiments were performed using deionized water from an in-house Milli-Q system (18.2 MΩ cm).

### 2.2 Synthesis of the mesoporous metal oxides

A sol–gel method extracted from Xaba *et al.*^26^ was used to synthesize the various metal oxide materials. Approximately 10 g of the P123 surfactant and 20 g of the metal precursor were dissolved with 60 mL butanol in a beaker. The mixture was agitated at room temperature until completely dissolved, and then 6.7 mL of 70% nitric acid was added and stirred overnight. To evaporate the solvent, the mixture was baked in the oven at 100 °C for 4 hours. The resulting precipitate was then washed with ethanol and dried at 60 °C overnight. Finally, the resulting powder was calcined at 350 °C for 6 hours.

### 2.3 Immobilization of Pd nanoparticles onto mesoporous metal oxide

The deposition–precipitation method was used to immobilize the palladium nanoparticles on metal oxide supports.^27^ The goal was to deposit 1 mol% palladium particles on each support. As a result, for all samples, 0.05 g potassium tetrachloropalladate and 4.95 g metal oxide support were suspended in 80 mL deionized water and agitated at room temperature for 30 min. The mixture was agitated for a further 2 hours at 80 °C, with urea added in a mole ratio of 1 Pd : 15 urea. After allowing the mixture to cool to room temperature, a 15 molar excess of sodium borohydride was added dropwise and stirred overnight. The resultant solution was filtered, rinsed with deionized water and then dried overnight in a vacuum oven at 80 °C. After that, the sample was crushed to a fine powder.

### 2.4 Catalyst characterization

On a Micromeritics ASAP 2460 sorption machine, nitrogen sorption measurements were taken at −196 °C using the Brunauer–Emmett–Teller (BET) method to quantify the materials' specific surface area, pore volume, and average pore diameter. Before the analyses, the samples were degassed with flowing nitrogen gas for 8 hours at 90 °C using a Micromeritics FlowPrep 060 sample degas system. To investigate the crystalline phases of the catalysts, an X'Pert Pro Powder X-ray diffractometer equipped with a Cu Kα_1_ (*λ* = 0.1542 nm) radiation source was used to obtain the X-ray diffractogram at room temperature. With a step rate of 0.4° per min, wide-angle measurements were taken between 10° and 90° 2*θ* for 2 minutes. The HighScore software was used to match the obtained p-XRD patterns. The morphological studies of the samples were conducted using a JEOL-JEM-2100F transmission electron microscope (TEM) with a 200 kV accelerating voltage. Prior to analysis, samples were mixed with ethanol; one drop of the suspension was placed on a holey-carbon-coated Cu grid allowing the solvent to evaporate in air. The average particle size and the particle size distribution were estimated from the micrographs obtained. The surface of the catalyst was analysed using scanning electron microscopy (SEM). Fresh samples were carbon-coated on an aluminium stub with an Agor Turbo carbon coater before being analysed with a VEGA 3 TESCAN scanning electron microscopy and an Oxford energy dispersive X-ray (EDX) equipment at 20 kV. The thermal behaviour of different catalysts was verified using a thermogravimetric analyser (TGA) PerkinElmer STA 6000 at a 10 °C min^−1^ ramping rate from 25 to 600 °C under nitrogen flow at a 100 mL min^−1^ flow rate.

### 2.5 Catalytic studies

#### 2.5.1 Buchwald–Hartwig amination

An amine (1.2 equiv., 1.2 mmol), an aryl halide (1 equiv., 1 mmol) and a catalyst (50 mg, 1 mol% Pd) were transferred into a vial. Then, 5 mL of the 2 wt% HPMC aqueous solution and a base (2 equiv., 2 mmol) were added and stirred. Upon completion of the reactions, extraction of products was done with 3 × 5 mL ethyl acetate. Anhydrous magnesium sulphate was used as a drying agent. Purification of the product was performed through a silica-packed column.

#### 2.5.2 Sonogahira coupling

An amine (1.2 equiv., 1.2 mmol), an alkyne (1 equiv., 1 mmol) and a catalyst (50 mg, 1 mol% Pd) were transferred into a vial. Then, 5 mL of the 2 wt% HPMC solution and a base (2 equiv., 2 mmol) were added and stirred. Upon completion of the reactions, extraction of products was done with 3 × 5 mL ethyl acetate. Further drying of the product was done with magnesium sulphate. Purification of the product was done through silica packed column.

Both reactions were monitored with GC-FID and GC-MS. A Shimadzu GC-2010 equipped with a flame ionization detector (FID) and a Restek-800-356-1688 capillary column (30 m × 0.25 mm × 0.25 m) was utilized for quantitative analysis, with injection port and FID temperatures of 250 °C and 300 °C, respectively. A GC-MS equipped with a capillary column and mass spectrometer was used to confirm the targeted products. The integrations obtained from the GC-FID data were used to compute substrate conversion, selectivity, and product yields. (See eqn (S2)–(S4) on ESI†). Furthermore, the structures of the products were matched from the GC software library. The ^1^H NMR (500 MHz) and ^13^C NMR (125 MHz) spectra were taken on a Bruker-500 MHz spectrometer, and the results were determined using tetramethylsilane (0.0 ppm) as the internal standard.

## 3. Results and discussion

### 3.1 Catalyst characterization

#### 3.1.1 TEM analysis

**Fig. 1:**
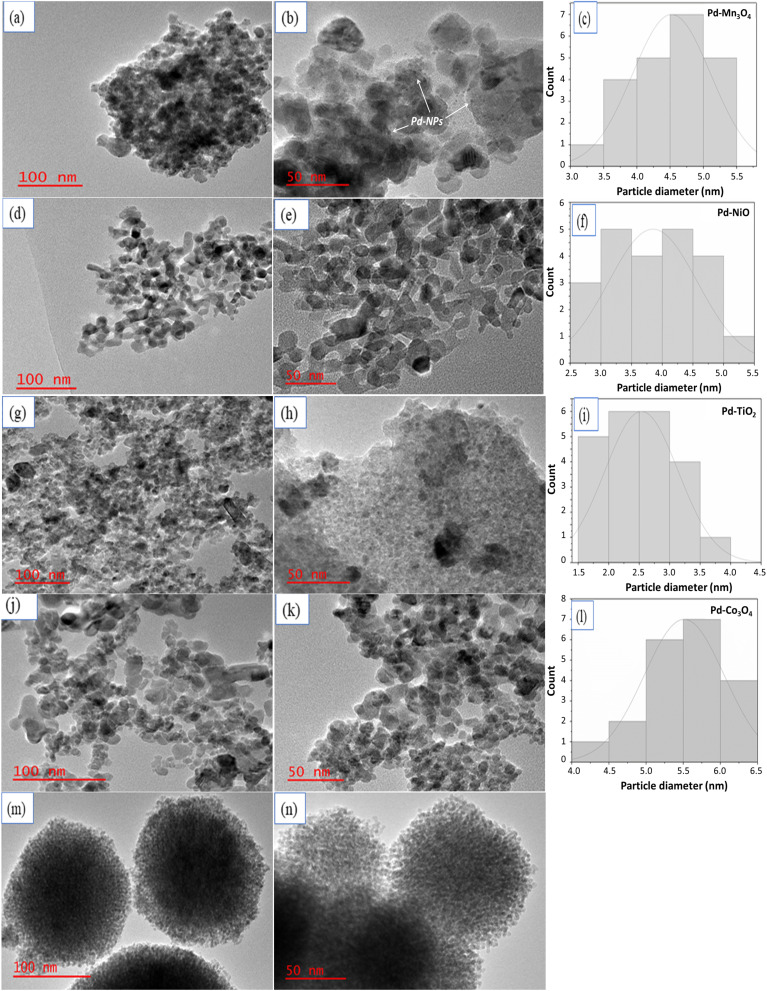
TEM images of Pd–Mn_3_O_4_ (a) and (b), Pd–NiO (d) and (e); Pd–TiO_2_ (g) and (h); Pd–Co_3_O_4_ (j) and (k); and Pd–CeO_2_ (m) and (n) with their corresponding particle size distribution histograms (c), (f), (i) and (l), respectively.

A high-magnification TEM was used to evaluate the morphology of the palladium-based synthesized catalysts. The representative TEM images (Fig. 1) show well-distributed nanoparticles on the porous surface of catalysts, and a clear network of pores is present in all the materials. The image in Fig. 1(m) shows CeO_2_ as small clusters with small uniform porous channels all over the surface of the catalyst; which then confirms that the surface is indeed porous. These observations align also with the nitrogen sorption results reported in this study. The average size of the palladium particles and the particle size distribution (see Fig. 1(c), (f), (i) and (l)) were estimated from the micrographs obtained using Image J software. Image J software uses a two-dimensional approach whereby the diameters of over 100 nanoparticles, assuming virtually spherical and randomly oriented were manually measured, and their data were collectively used for the construction of the size distribution histograms. Though metal nanoparticles are not clearly perceptible in supported catalysts due to the appearance features of the supports, selected palladium particles are highlighted in Fig. 1(b). The palladium nanoparticles are rather homogeneously distributed over the supports. It can be observed from the size distribution histograms that the sizes of the palladium nanoparticles for most of the catalysts are in the range of 1.5 nm to 6.5 nm, with the average size of 4.5 nm for Pd–Mn_3_O_4_, 3.8 nm for Pd–NiO, 2.6 nm for Pd–TiO_2_ and 5.5 nm for Pd–Co_3_O_4_. We could not estimate the sizes of the nanoparticles supported on CeO_2_ using Image J since the nanoparticles were embedded inside the pores and not clearly visible on the catalyst surface.

#### 3.1.2 SEM analysis

**Fig. 2:**
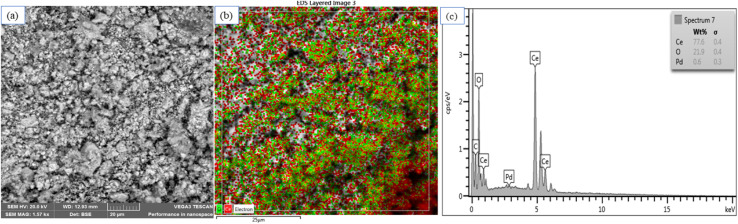
(a) SEM image of Pd–CeO_2_; (b) elemental mapping image of Pd–CeO_2_; (c) SEM-EDX image of Pd–CeO_2_.

The SEM image in Fig. 2(a) depicts the visual morphology of the surface of the catalyst. The image shows that there was no single shape but rather a wide range of shapes of the catalyst particles. The elemental mapping in Fig. 2(b) illustrates the distribution morphology of each element that makes up the catalyst. The signals of the SEM-EDX (Fig. 2(c)) are in line with the signals of XRD (see Fig. 4), showing high signal intensity for cerium oxide. Moreover, the EDX analysis revealed the presence of three elements, Ce, O and Pd as expected, with the loading of palladium being approximately 1%. The weight percentage of the elements (wt%) obtained confirmed the purity of the as-synthesized catalyst.

#### 3.1.3 Nitrogen sorption measurements

**Fig. 3:**
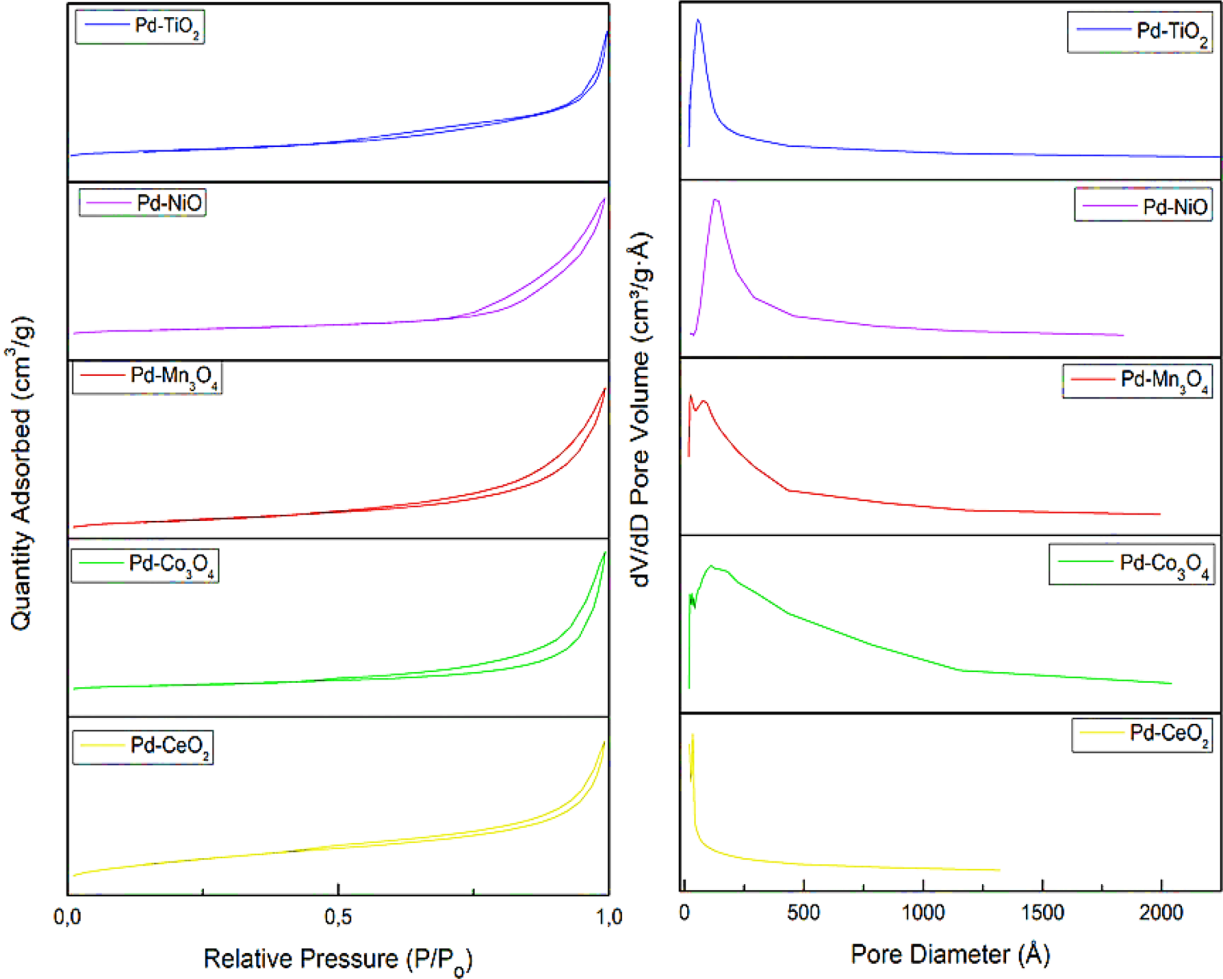
BET isotherms of various Pd-MMOs catalysts and their corresponding pore volume *vs.* pore diameter plots.

The N_2_-adsorption of the mesoporous oxide-supported palladium catalysts is illustrated in Fig. 3. All the catalysts, Pd–TiO_2,_ Pd–NiO, Pd–Mn_3_O_4_, Pd–Co_3_O_4_, and Pd–CeO_2_, show adsorption–desorption curves that represent type IV isotherms with hysteresis loops. This type of behaviour is an indication of the mesoporous nature of the material.

Another significant factor to investigate is the distribution of the pores in these metal oxides. The pore size distribution plots indicate that all the catalytic materials are within the mesoporous range, from 2 to 50 nm. It can be observed from Table 1 that the surface areas of all the catalysts are acceptably high, greater than 50 m^2^ g^−1^, and the pore sizes are less than 30 nm, signifying the mesoporous nature of the catalysts. The pore volumes are also notable, with Pd–NiO showing the lowest pore volume of 0.063 cm^3^ g^−1^.

**Table 1:** Nitrogen sorption results showing the surface area, pore diameter and pore volume of the mesoporous oxide-supported palladium catalysts

Sample	BET surface area (m^2^ g^−1^)	Pore diameter (nm)	Pore volume (cm^3^ g^−1^)
Pd–TiO_2_	89.78	14.32	0.1441
Pd–NiO	51.63	9.071	0.0626
Pd–Mn_3_O_4_	66.45	15.66	0.0919
Pd–Co_3_O_4_	62.64	26.87	0.0662
Pd–CeO_2_	84.11	23.11	0.1760

#### 3.1.4 The p-XRD analysis

**Fig. 4:**
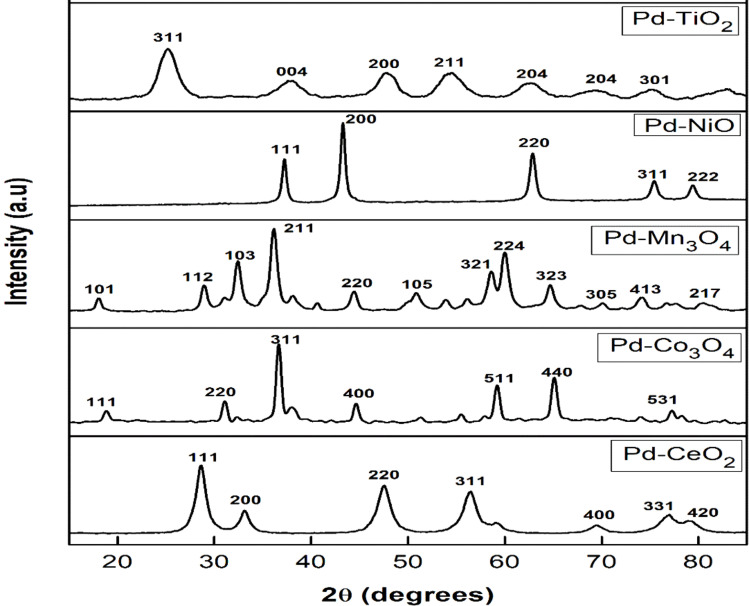
p-XRD patterns of Pd–TiO_2_, Pd–NiO, Pd–Mn_3_O_4_, Pd–Co_3_O_4_ and Pd–CeO_2_ at wide angles.

The wide-angle XRD patterns of all the mesoporous oxides supported palladium catalysts are displayed in Fig. 4. The characteristic peaks of the synthesized TiO_2_ are seen at 25° to 55° for the wide-angle analysis, with an intense peak around 26° indicating the anatase phase of the TiO_2_. All the catalysts somewhat lack long-range order; they showed an amorphous X-ray diffraction pattern at the lower angle, also indicating a mesoporous nature of the synthesized materials.

From the HighScore software, JCPD reference # 01-071-1168 revealed that the synthesized Pd–TiO_2_ is in the anatase phase, with a tetragonal crystal system and the space group *I*4_1_/*amd*. JCPD reference # 04-003-5840 revealed that the halite Pd–NiO has a cubic crystal system and that the space group is *Fm*3̄*m*. From the JCPD reference # 04-007-1841, it was found that the halite Pd–Mn_3_O_4_ has a tetragonal crystal system and the space group of *I*4_1_/*amd*. JCPD reference # 01-078-5620 revealed that the halite Pd–Co_3_O_4_ has a cubic crystal system, and the space group is *Fd*3̄*m*. Finally, JCPD reference # 04-018-6807 revealed that the halite Pd–CeO_2_ has a cubic crystal system, and the space group is *Fm*3̄*m*. As expected, all the catalysts illustrated a single crystalline phase structure, indicating the successful synthesis of the materials. Scherrer's equation (see eqn (S1)†) was used to estimate the crystallite sizes of the materials. The supported TiO_2_ catalyst exhibited a greater crystallite size of 102.48 nm compared to its counterparts, with crystallite sizes of less than 50 nm. This could be attributed to the difference in the ionic radii of their most stable oxidation states (Table 2).

**Table 2:** p-XRD data of the palladium-based catalysts, including crystallite sizes, space groups, and crystal system

Sample	Crystallite sizes^a^ (nm)	Space group	Crystal system
Pd–Co_3_O_4_	33.83	*Fd*3̄*m*	Cubic
Pd–Mn_3_O_4_	49.21	*I*4_1_/*amd*	Tetragonal
Pd–NiO	20.62	*Fm*3̄*m*	Cubic
Pd–CeO_2_	47.15	*Fm*3̄*m*	Cubic
Pd–TiO_2_	102.5	*I*4_1_/*amd*	Tetragonal

a Calculated using Scherrer's equation.

#### 3.1.5 Thermogravimetric analysis

A highly thermally stable support is of good choice for many catalytic applications, as it maintains a high specific surface area at high temperatures, and consequently maintains high dispersion of nanoparticles. The thermal stability graph of the Pd–CeO_2_ is reported in (Fig. S1†). The plot shows a gradual decrease from 25 °C to 800 °C. The weight loss at a lower temperature, less than 100 °C, corresponds to the loss of absorbed water from the catalysts, while the continuous weight loss above 100 °C could be assigned to the decomposition of the reducing agent chemisorbed onto the catalyst's surface. The weight loss of up to 7.5% observed confirms the thermal stability of the catalysts at temperatures up to 800 °C.

### 3.2 Buchwald–Hartwig aminations

#### 3.2.1 Catalyst screening

Various transition metal oxides supported palladium nanoparticles were screened for Buchwald–Hartwig amination application. The catalysts that showed promising activity are reported in Table 3. A Buchwald–Hartwig coupling of 4-bromotoluene and aniline to yield a product called 4-methylbiphenylamine was chosen as a model reaction for the catalytic studies. The reactions were monitored hourly for 4 hours and then left running overnight. We observed that the conversions increased as the time increased (see Fig. S2†); for example, the Pd–CeO_2_ completed a 100% conversion of the aryl halide after three hours of the reaction and remained at 100% throughout the last hour of screening.

**Table 3:** The yield, conversion and selectivity obtained for each reaction during the catalyst screening study

Entry	Catalyst	GC yield (%)	Conversion (%)	Selectivity (%)
1	Pd–Co_3_O_4_	24	30	50
2	Pd–Mn_3_O_4_	82	88	100
3	Pd–NiO	20	32	33
4	Pd–CeO_2_	97	100	100
5	Pd–TiO_2_	61	65	100

Table 3 and Fig. S3† additionally showed the comparison by GC yield of the 4-methylbiphenylamine produced when Pd–NiO, Pd–Co_3_O_4_, Pd–Mn_3_O_4_, Pd–TiO_2_ and Pd–CeO_2_ were used as catalysts for the model reaction. Amongst all the evaluated catalysts, the Pd–CeO_2_ produced the highest yield (97%) of the 4-methylbiphenylamine with a 100% selectivity to only one product. The Pd–CeO_2_ was then used for substrate screening and other studies. The least active catalyst was Pd–NiO which yielded only 20% of the 4-methylbiphenylamine, with 32% conversion and 33% selectivity.

The high catalytic performance over Pd–CeO_2_ can be attributed to the synergistic effect of Pd and CeO_2_. It was reported that the interaction between Pd and CeO_2_ plays a crucial role in enhancing the catalytic performance of Pd–CeO_2_.^28^ The ceria supported palladium catalysts, particularly single-site Pd_1_–CeO_2_, have shown remarkable performance in Buchwald–Hartwig amination reactions, especially in the selective amination of phenols to aromatic amines without external hydrogen sources.^28,29^ The unique properties of CeO_2_, such as its ability to stabilize single-site Pd and facilitate specific reaction pathways, contribute to this enhanced catalytic performance. Wang *et al.* reported on thermally-stable single-site Pd_1_–CeO_2_ catalyst for selective amination of phenols to aromatic amines.^29^ The obtained Pd_1_–CeO_2_ catalysts achieved remarkable selectivity of important aromatic amines (up to 76.2% yield) in the phenols amination with amines without external hydrogen sources, while traditional Pd nanoparticles mainly favoured the formation of phenyl-ring-saturation products. These results indicated that our in-house synthetized Pd–CeO_2_ catalyst shows an excellent catalytic activity in Buchwald–Hartwig amination reactions, enabling the formation of the desired aromatic amines.

#### 3.2.2 Reaction parameters

To investigate the optimal conditions, the reaction of 4-bromotoluene with aniline, catalyzed with Pd–CeO_2,_ was selected as a model reaction. The model reaction was used to study the effects of various reaction parameters, including reaction times, bases, solvents, and temperature. The results of optimization studies are shown in Table 4, where the reaction was monitored hourly for 6 hours to also observe the stability of the catalyst and the effects of time on the reaction.

**Table 4:** Evaluation of the optimal reaction parameters, including bases and temperature^a^

Entry	Catalyst	Temperature (°C)	Base	GC yield (%)	Conversion (%)	Selectivity (%)
1	No catalyst	80	K_2_CO_3_	No reaction	No reaction	No reaction
2	CeO_2_	80	K_2_CO_3_	No reaction	No reaction	No reaction
3	Pd–CeO_2_	25	K_2_CO_3_	24	30	100
4	Pd–CeO_2_	50	K_2_CO_3_	60	63	100
5	Pd–CeO_2_	80	K_2_CO_3_	97	100	100
6	Pd–CeO_2_	80	KOH	61	70	100
7	Pd–CeO_2_	80	NEt_3_	23	32	80
8	Pd–CeO_2_	80	Cs_2_CO_3_	55	60	33
9	Pd–CeO_2_	80	Na_2_CO_3_	63	66	100

a Reaction conditions: aniline (1.2 equiv., 1.2 mmol), 4-bromotoluene (1 equiv., 1 mmol), catalyst (50 mg) and base (2 equiv., 1.5 mmol), 6 h.

The evaluation of the optimum temperature began at room temperature, with the optimal temperature discovered at 80 °C. This temperature is still considered mild and fits with the motive of the project. Moreover, various bases were investigated for this study, including caesium carbonate, sodium carbonate, potassium carbonate, potassium hydroxide, and triethylamine. Potassium carbonate was found to be the best base, with the reaction yielding 97% of the product, and 100% conversion and selectivity. Entries 1 and 2 in Table 4 represent the control experiments. In the absence of a catalyst, there was no reaction observed. Also, when the CeO_2_ support (without palladium nanoparticles) was used to catalyse the Buchwald–Hartwig aminations, no conversion of the aryl halide occurred, thus no reaction happened. It is therefore postulated that the Buchwald–Hartwig reaction in our system is driven by the palladium nanoparticles. However, we supported the nanoparticles on high surface area solid supports for better stability, good dispersion and durability.^18^

#### 3.2.3 Substrate study

The substrate study was conducted to investigate the generality of the Buchwald–Hartwig amination reactions by using various aryl halides with various functional groups. Most substrates used in the Buchwald–Hartwig amination study were insoluble in water alone, but their solubility improved in the presence of HPMC. The functional groups represented electron-withdrawing, electron-donating and sterically bulky groups which had various effects on the outcome of the amination reactions. Some of the products in Table 5 (entries 8, 11, 12 and 14) were produced with lower yields due to their corresponding starting materials' low solubilities in water, even with the presence of HPMC. However, most products were obtained with high yields ranging from 71% to 100%. The table (entries 1 and 10) indicates that the yield of the products obtained when benzylic halide was used as substrate is reported at 100% and 97% respectively, and is higher than those obtained when the aryl halide substrates were used (other entries). Benzylic halides (sp^3^ hybridization) are generally more reactive than aryl halides (sp^2^ hybridization) due to the resonance stabilization of the carbocation formed during the reactions.

**Table 5:** The yields and selectivity of various products formed for the Buchwald–Hartwig amination of benzamine with various aryl halides substrates^a^

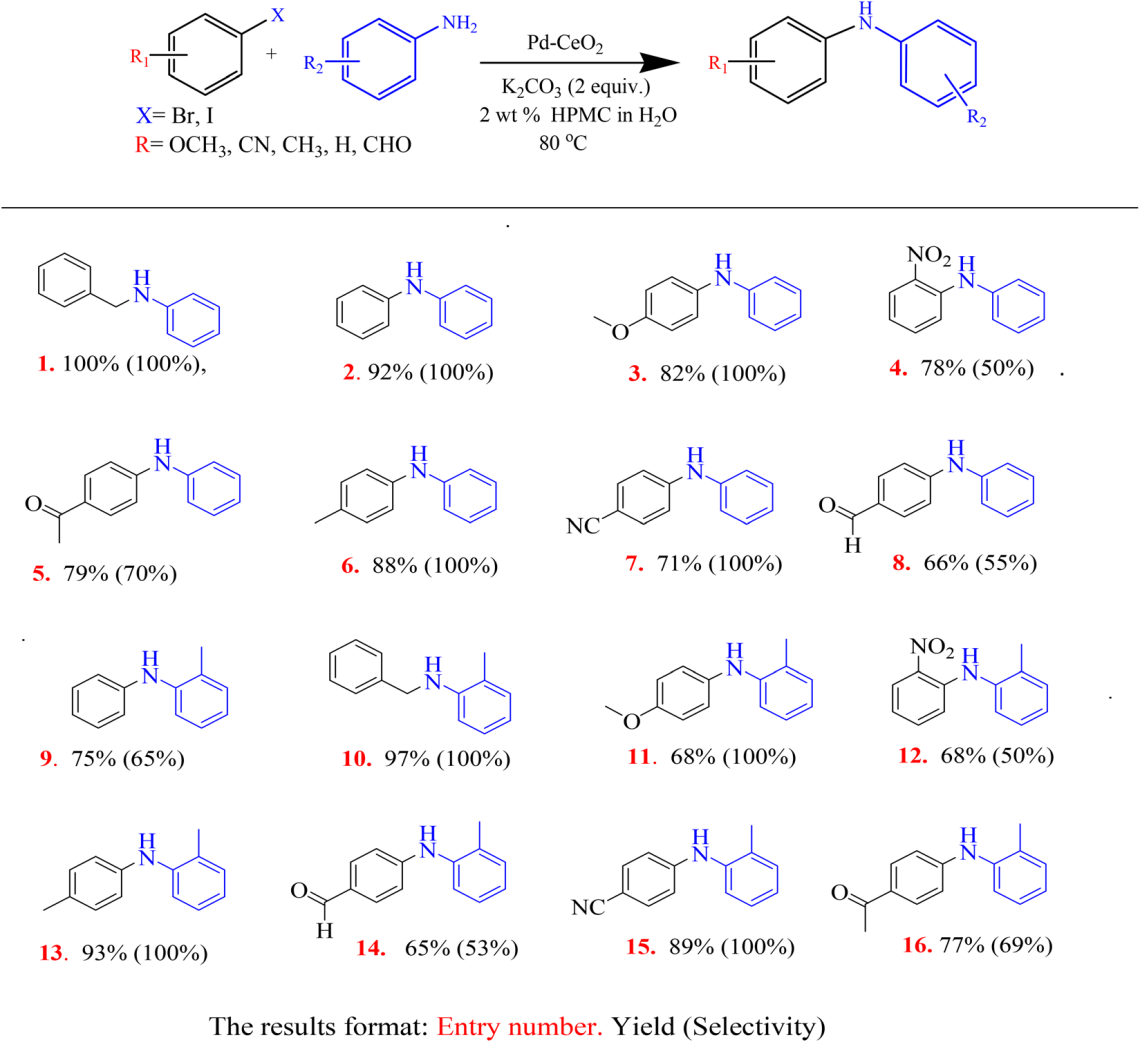

a Reaction conditions: amine (1.2 equiv., 1.2 mmol), aryl/benzylic halide (1 equiv., 1 mmol), Pd–CeO_2_ (50 mg) and K_2_CO_3_ (2 equiv., 2 mmol) at 80 °C.

With the unsubstituted benzamine (aniline), the non-functionalized aryl halide (entry 2) led to a better yield of the product (92%) compared to the functionalized aryl halides (entries 3 to 8). The aryl halide substrates containing electron-donating groups, such as methoxy (entry 3) and *para*-methyl (entry 6) were proven to be compatible with the reaction and led to excellent yields, 82% and 88% respectively. However, the electron-withdrawing groups (entries 4, 5 and 7) led to good yields, greater than 70% (see Table 5). With the substituted benzamine (*o*-toluidine), the aryl halide containing a *para*-methyl electron-donating group (entry 13) reacted well with a high yield of 93% being obtained, followed by the aryl halide with a cyano electron-withdrawing group, 89% (entry 15).

### 3.3 Sonogashira coupling

The performance of the prepared catalysts on Sonogashira coupling involved the same set of palladium-supported catalysts used for Buchwald–Hartwig amination. To achieve optimal conditions, the reaction of 4-bromoacetophenone with phenylacetylene was selected as a model reaction. During the catalyst screening study (Table 6), the model reaction was monitored with each catalyst hourly for 6 hours and then left running overnight. The conversions increased as the time increased; the most active catalyst, Pd–Co_3_O_4_ completed the reaction at 2 hours and remained at 100% conversion until the 6th hour. The optimal working temperature was found to be 80 °C and the results are tabulated in Table 6.

**Table 6:** The yields, conversions, and selectivity obtained for each reaction during the catalyst screening study for Sonogashira coupling reactions^a^

Entry	Catalyst	Conversion (%)	Selectivity (%)	GC yield (%)
1	Pd–Co_3_O_4_	100	100	90
2	Pd–Mn_2_O_4_	88	100	88
3	Pd–NiO	32	50	30
4	Pd–CeO_2_	61	100	60
5	Pd–TiO_2_	58	100	58

a Reaction conditions: phenylacetylene (1.2 equiv., 1.2 mmol), 4-bromoacetophenone (1 equiv., 1 mmol), catalyst (50 mg) and K_2_CO_3_ (2 equiv., 1.5 mmol), 80 °C, 6 h.

The substrate study reported in Table 7 was performed to monitor the general applicability of the Pd–Co_3_O_4_ catalyst for Sonogashira coupling. Interestingly, the reactions were successful and proceeded very smoothly without the addition of copper as a co-catalyst. Moreover, all the evaluated functional groups of the aryl halides were well tolerated and led to moderate to excellent yields, with the *para*-methyl electron-donating group (entry 1) reacting well with a product yield of 91%. As expected, the very active aryl, iodide formed a better yield than their aryl bromide counterpart (see entry 4). Aryl bromides are known to have intermediate activity between the aryl iodides and the slightly active aryl chlorides. Aryl iodides are generally more reactive than their aryl halogens counterparts due to their weaker C–I bond, making them easier to break and facilitating reactions like cross-coupling.

**Table 7:** The yields and selectivity of various products formed through combinations of various substituted aryl halides with phenylacetylene for the Sonogashira coupling^a^

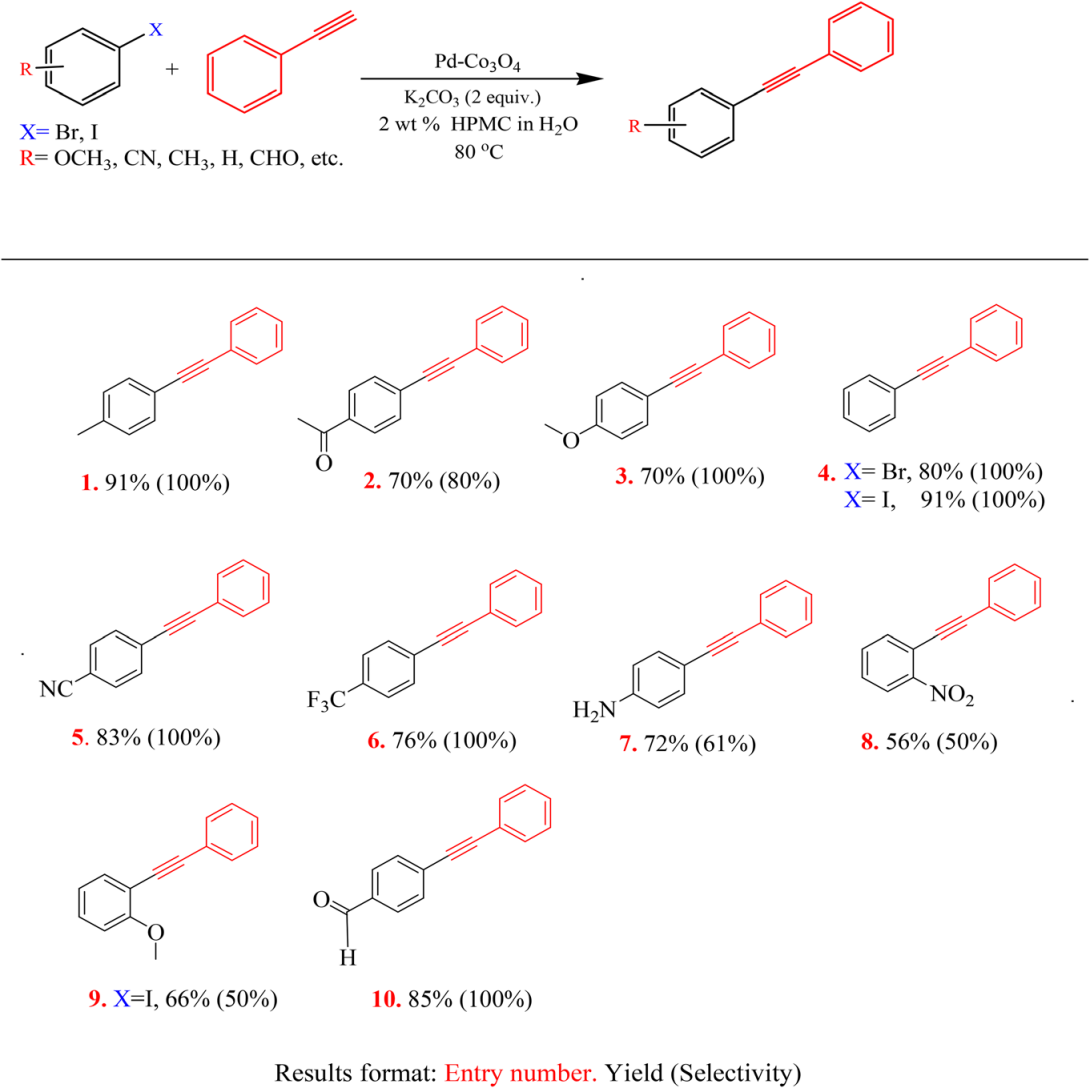

a Reaction conditions: phenylacetylene (1.2 equiv., 1.2 mmol), aryl halide (1 equiv., 1 mmol), Pd–Co_3_O_4_ (50 mg) and K_2_CO_3_ (2 equiv., 2 mmol) at 80 °C.

The high performance of Pd–Co_3_O_4_ catalysts compared to its counterparts can be attributed to the synergistic effect between Pd and Co_3_O_4_. The interaction between Pd and Co_3_O_4_ can lead to changes in the electronic structure of palladium; metal Pd nanoparticles adhered to the surface of Co_3_O_4_, and Pd gave electrons to Co_3_O_4_, which stabilized the catalyst and potentially affecting its reactivity.^30^ The Sonogashira reaction typically involves oxidative addition of the aryl or vinyl halide to Pd(0), followed by transmetallation, and then reductive elimination to form the new C–C bond. The presence of Co_3_O_4_ can influence these steps, potentially accelerating the overall reaction.^31^ Kakanejadifard *et al.* reported that Pd–Co_3_O_4_ catalysts, particularly in the form of nanoalloys, can be effective in Sonogashira coupling reactions, offering advantages like high activity and recyclability.^32^ The formation of Pd–Co_3_O_4_ nanoalloys involves dispersing metal nanoparticles on the support, creating a structure where Pd and Co_3_O_4_ components interact at the nanoscale. Generally, in the conventional Sonogashira coupling, palladium acts as the primary catalyst, while copper often serves as a co-catalyst, facilitating the activation of the alkyne. When present in a nanoalloy structure, palladium and copper can exhibit a synergistic effect, which notably enhance the reaction rate.^31–33^

The plausible structures of the products were matched from the GC software library, and a number of GC-MS spectra are displayed in Fig. S4.† The formation of the products for Sonogashira coupling and Buchwald–Hartwig amination reactions was further confirmed by ^1^H NMR and ^13^C NMR. The substrate study products and their corresponding spectra are presented in Fig. S5 and S6,† respectively.

### 3.4 The catalyst reusability studies

**Fig. 5:**
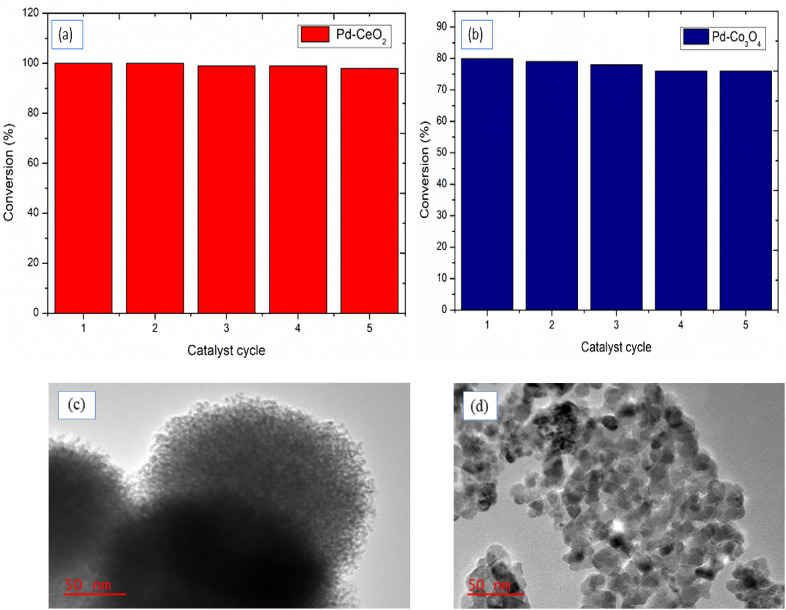
Recyclability studies of (a) Pd–CeO_2_ for Buchwald–Hartwig amination and (b) Pd–Co_3_O_4_ for Sonogashira coupling; TEM images of recycled (c) Pd–CeO_2_ and (d) Pd–Co_3_O_4_.

The stability of solid catalysts depends on their long-term durability without significant loss in reactivity in terms of activity and selectivity. The recycling study of the catalysts was performed under the optimized reaction conditions for both Buchwald–Hartwig coupling and Sonogashira coupling. Fig. 5(a) and (b) illustrates the reusability of the catalysts up to the fifth run.

Mesoporous Pd–CeO_2_ was used five consecutive times in the recyclability test for Buchwald–Hartwig amination using the coupling of bromobenzene and aniline as a model reaction. After each run, the catalyst was isolated through simple centrifugation, washed several times with deionised water, and then dried and weighed. The conversions remained high, at 98% after five cycles. Similarly, the Pd–Co_3_O_4_ catalyst was recycled in the application of Sonogashira coupling of bromobenzene and phenylacetylene reactions. The Pd–Co_3_O_4_ maintained its stability, and the conversions remained within a 5% difference for the five cycles. It can be also observed from Fig. 5(c) and (d) from the TEM images that the surface morphology and porous nanostructure of the catalysts was not significantly changed after fifth run, again showing the stability of the catalyst. These features make them good catalysts in both academic research and industrial applications.

## 4. Conclusions

In this study, palladium nanoparticles were successfully immobilized on the solid supports, and utilized as catalysts for the Buchwald–Hartwig amination and the Sonogashira coupling reactions. The characterisation studies showed that the sol–gel method and the deposition precipitation method used in this study, respectively, resulted in the formation of ordered mesoporous metal oxides and mono-dispersed palladium nanoparticles with narrow size distributions. We have demonstrated that the mesoporous materials supported by Pd nanocatalysts are highly efficient for the C–N and C≡C coupling reactions in water. The Pd–CeO_2_ was found to be the most active catalyst of all studied catalysts, and K_2_CO_3_ was the optimum base for the Buchwald–Hartwigs amination reaction. The Sonogashira coupling reactions were quite successful, as Pd–Co_3_O_4_ emerging as the best catalyst. Various functional groups of the aryl halides were well tolerated and led to alkyne products with high yields and selectivity. The reusability studies indicated that both The Pd–CeO_2_ and Pd–Co_3_O_4_ catalysts can be recovered and reused with retained performances for up to at least five consecutive reaction cycles.

## Conflicts of interest

The authors declare no conflict of interest.

## Supplementary Material

RA-015-D5RA02824H-s001

## Data Availability

The data supporting this article have been included as part of the ESI.†
