# Supplementary material for: Application of palladium nanoparticles supported on ordered mesoporous oxides for C–N and C[triple bond, length as m-dash]C coupling reactions in water
Source: RSC Adv. 2025 Jul 29;15(33):26981–91. doi: 10.1039/d5ra02824h (PMC12305527; doi:10.1039/d5ra02824h)
Supplement: RA-015-D5RA02824H-s001 [file RA-015-D5RA02824H-s001.pdf]

Supplementary Information (SI) for RSC Advances.
This journal is © The Royal Society of Chemistry 2025

Application of Palladium Nanoparticles Supported on Ordered Mesoporous Oxides for C−N and C≡C Coupling Reactions in Water

Nomathamsanqa Prudence Maqunga, Matumuene Joe Ndolomingo, Ndzondelelo Bingwa, and Reinout Meijboom*

Department of Chemical Sciences; University of Johannesburg; PO Box 524; Auckland Park; Johannesburg 2006; South Africa.

*Tel.: +27 (0)11 559 2367, fax: +27 (0)11 559 2819; *e-mail:* rmeijboom@uj.ac.za

1. Supplementary Information

1. Crystallographic data

Debye-Scherrer's equation: $$D = \frac{k\lambda}{\beta cos\theta}$$ Equation S1

Where D is the particle size (nm), k = 0.89 (dimensionless shape factor), λ is the X-ray wavelength, β is the line broadening at half the maximum intensity (FWHM) and θ is the angle derived from 2θ values corresponding to maximum intensity peak in the p-XRD diffractogram.

2. Thermogravimetric analysis

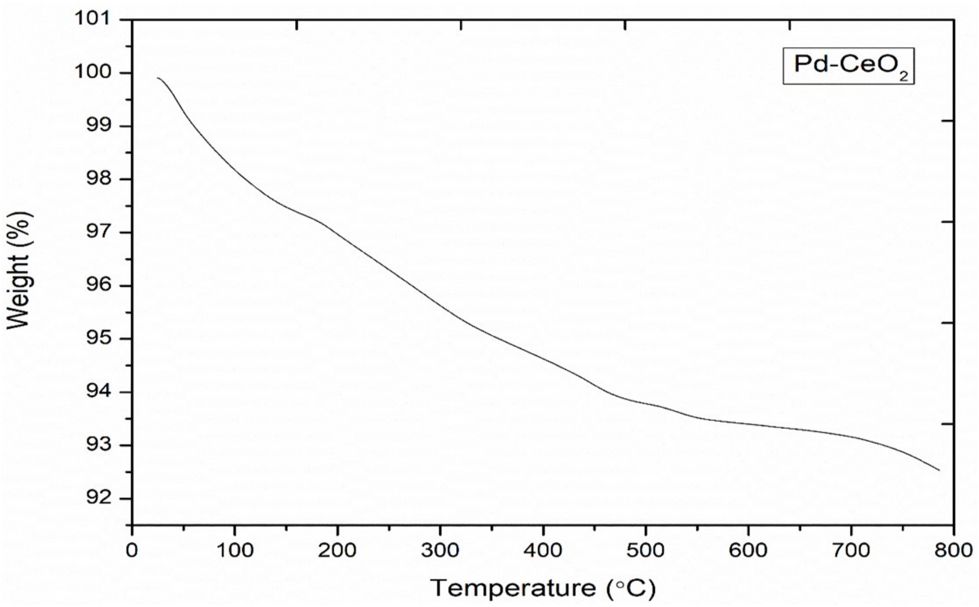

Figure S1: Thermal gravimetric analysis plot of the in-house synthesized Pd-CeO_2 analysed at 10 °Cmin^{-1} ramping rate from 25–800 °C.

3. Preparation of 2% HPMC stock solution

Millipore water (60 mL) was heated to 70 °C while stirring. Then 2 g of HPMC was added and stirred until dissolved. Finally, 40 mL of Millipore water was added, to obtain a 2 wt. % HPMC solution, and the mixture was allowed to cool to room temperature.

4. GC-FID methods

The Shimadzu 2010 Plus GC is equipped with a split/splitless inlet using a Restek RTX-5 (5% diphenyl/95% dimethylpolysiloxane) column (30 m x 0.25 mm x 0.25 µm). The carrier gas was Nitrogen gas at a flow rate of 1.82 mL/min. Samples (1 µL) were injected with a split ratio of 20:1 at 200 °C. The temperature was constant at 40 °C for 2 min then increased to 280 °C, at speed of 10 °C/min, and then kept constant for 5 min. A flame ionization detector (FID) was used at 350 °C.

Conversion percentage $= \left[1 - \frac{A_t}{I_t} \Big/ \frac{A_0}{I_o}\right] \times 100$ Equation S2

Selectivity percentage $= \frac{Product_{\ a}}{product_{\ a} + product_{\ b} + product_{\ c}} \times 100$ Equation S3

Yield percentage $= \left[\frac{conversion}{100}\right] \times \left[\frac{selectivity}{100}\right] \times 100$ Equation S4

5. Catalyst screening for Buchwald-Hartwig amination reaction.

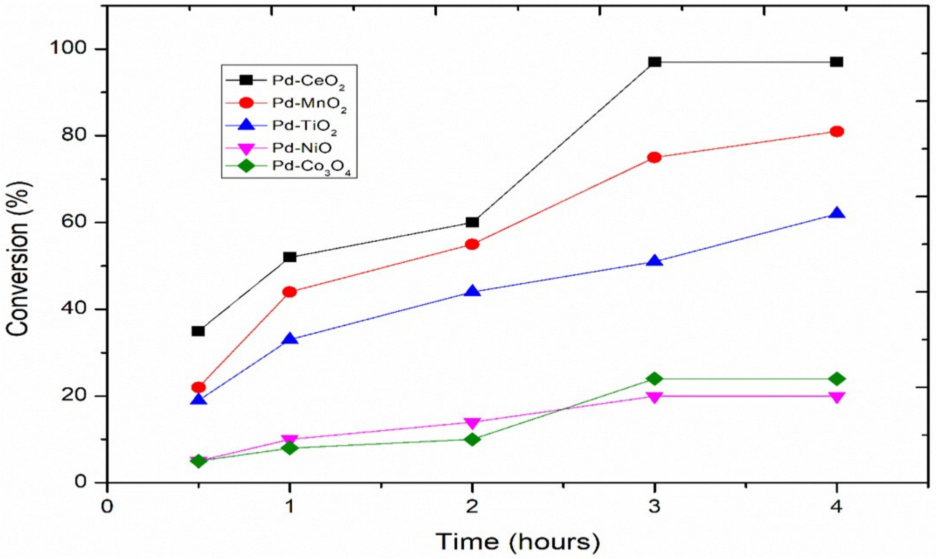

Figure S2: Effect of time on the percentage conversion for the Buchwald-Hartwig amination reaction using various catalysts.

6. Buchwald-Hartwig amination reaction using various catalysts

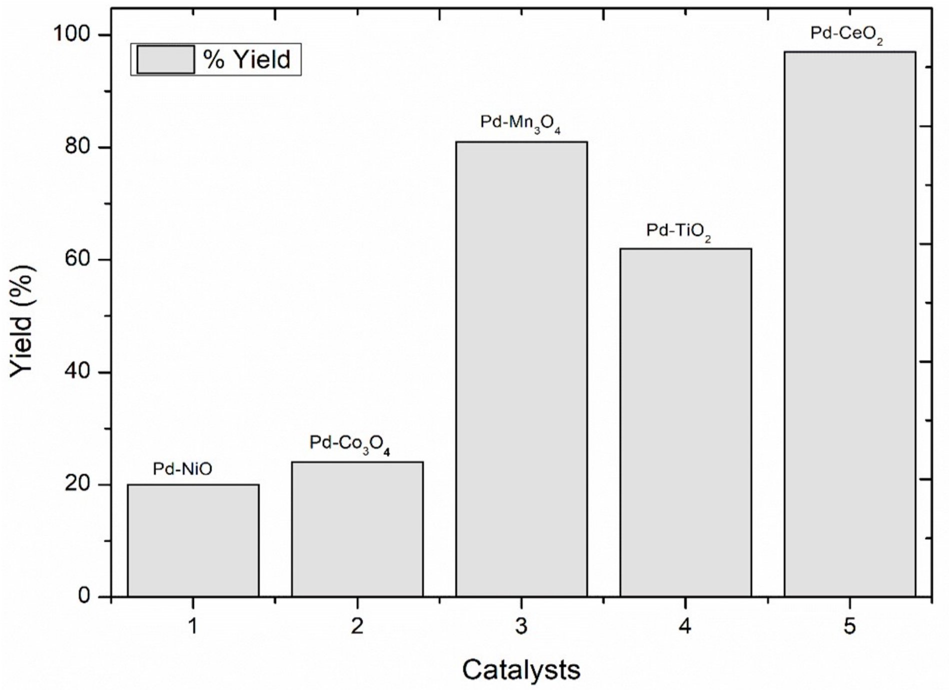

Figure S3: Yields obtained when various supported palladium catalysts were used for Buchwald-Hartwig amination reaction.

7. GC-MS results

1. 2-Nitro-diphenylamine

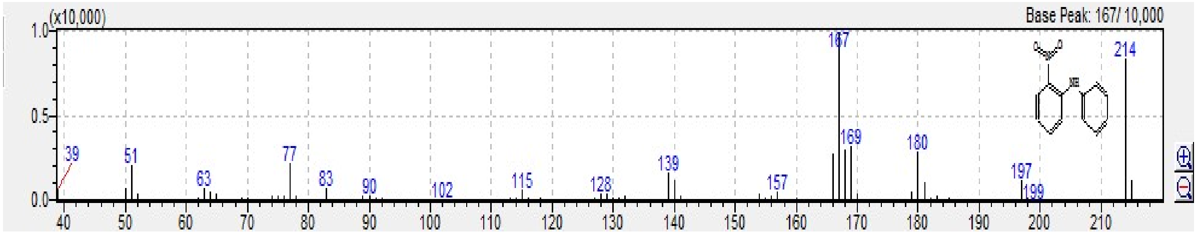

2. N-benzylidene

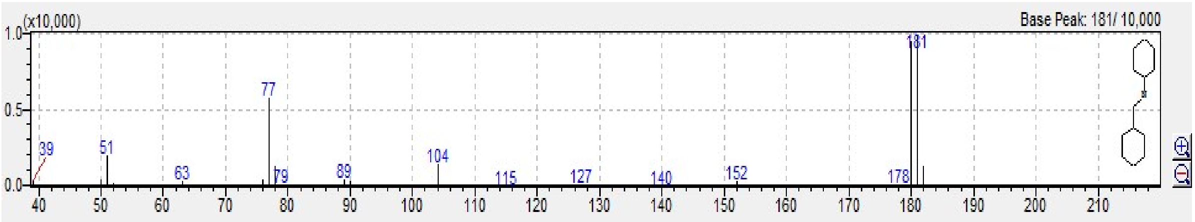

3. 1- (4-ethanone-phenyl)-phenylamine

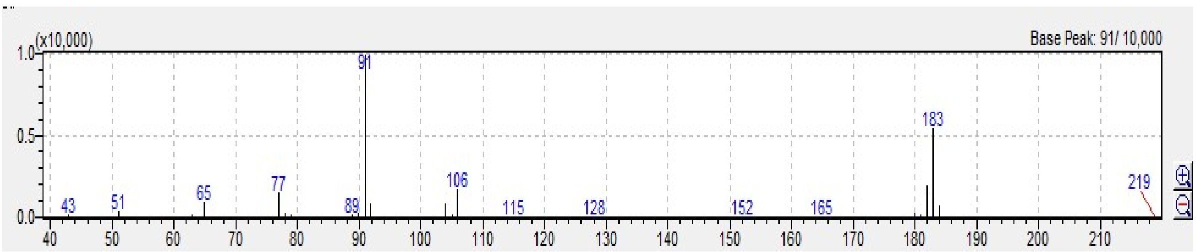

4. 1-(4-methoxy-phenyl)-phenylamine

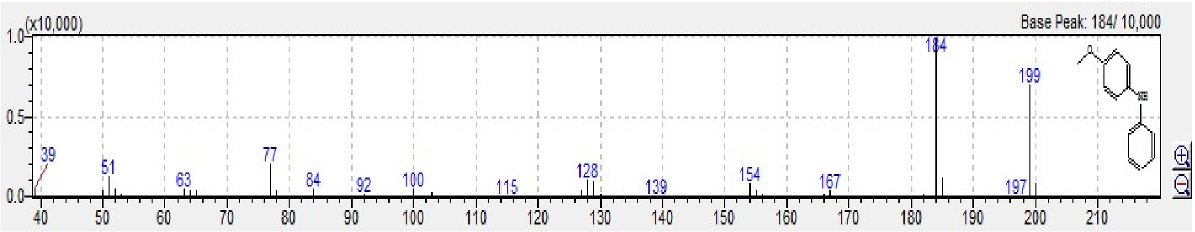

5. Biphenyl-amine

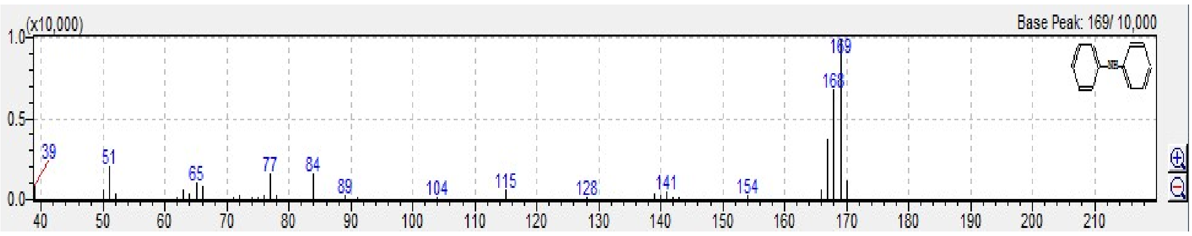

Figure S4: The GC-MS spectra of the plausible products obtain during the coupling reactions.

8. ^{1}H NMR and ^{13}C NMR for Sonogashira and Buchwald-Hartwig coupling products

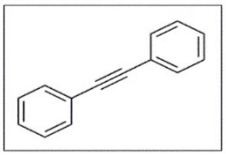

1,2-Diphenylethyne [6,7]
^{1}H NMR (500 MHz, $CDCl_3$) δ (ppm) = 7.62 –7.44 (m, 4H), 7.43 – 7.38 (m, 6H). **^{13}C NMR (125 MHz, $CDCl_3$) δ (ppm)** = 131.66, 128.46, 128.31, 123.31, 89.46.

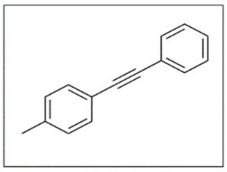

1-Methyl-4-(phenylethynyl) benzene [4,6,7]

^{1}H NMR (500 MHz, $CDCl_3$) δ (ppm) = 7.57 – 7.46 (2H, m), 7.40 – 7.38 (5H, m), 7.38 – 7.18 (2H, m), 2.42 (3H, s). **^{13}C NMR (125 MHz, CDCl3) δ (ppm)** = 138.16, 131.26, 129.37, 128.29, 128.03, 123.52, 123.49, 120.20, 90.49, 88.73, 21.46.

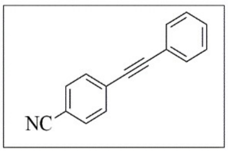

4-(Phenylethynyl) benzonitrile [3]
^{1}H NMR (500 MHz, $CDCl_3$) δ (ppm) = 7.68 – 7.60 (4H, m), 7.59 – 7.43 (2H, m), 7.42 – 7.29 (3H, m). **^{13}C NMR (125 MHz, CDCl3) δ (ppm)** = 132.11, 131.77, 129.28, 128.66, 128.23, 122.24, 118.58, 111.50, 93.81, 87.75, 77.37.

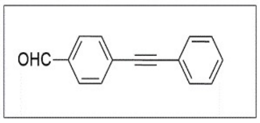

4-(Phenylethynyl) benzaldehyde [6] [3]
^{1}H NMR (500 MHz, CDCl3) δ (ppm) = 10.21 (1H, s), 7.89 (2H, d, *J* = 8.5 Hz), 7.70 (2H, d, *J* = 8.6 Hz), 7.60 – 7.58 (m, 2H), 7.42 – 7.30 (m, 3H). **^{13}C NMR (125 MHz, CDCl3) δ (ppm) =** 189.94, 135.41, 132.18, 131.82, 131.62, 129.56, 129.05, 128.46, 122.53, 93.51, 88.60, 77.52.

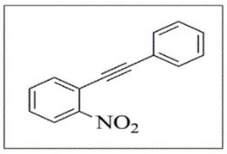

1-Nitro-2-(2-phenylethynyl) benzene [4,6]
^{1}H NMR (500 MHz, CDCl3) δ (ppm) = 8.13 (1H, d, *J* = 8.3 Hz), 7.77 (1H, d, *J* = 8.6 Hz), 7.65 – 7.64 (3H, m), 7.63 (1H, t, *J* = 9.5.0 Hz), 7.52 – 7.30 (3H, m). **^{13}C NMR (125 MHz, CDCl3) δ (ppm) =** 149.55, 134.53, 132.77, 131.97, 129.19, 128.55, 128.41, 124.68, 122.33, 118.72, 97.09, 84.71, 77.30.

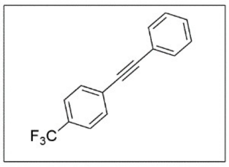

1-(Phenylethynyl)-4-(trifluoromethyl) benzene [3,6]
^{1}H NMR (500 MHz, CDCl3) δ (ppm) = 7.65 (4H, q, *J* = 11.5 Hz), 7.58 – 7.56 (2H, m), 7.39 – 7.37 (3H, m). **^{13}C NMR (125 MHz, CDCl3) δ (ppm) =**131.77, 131.72, 129.99, 129.67, 128.81, 128.43, 127.07, 125.24, 122.51, 91.72, 87.95.

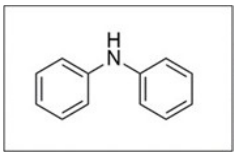

Diphenylamine [8]
^{1}H NMR (500 MHz, $CDCl_3$) δ (ppm) = 7.25 (4H, t, *J* = 8.0 Hz), 7.06 (4H, d, *J*= 7.2 Hz), 6.91 (2H, t, *J* = 7.3 Hz). **^{13}C NMR (125 MHz, $CDCl_3$) δ (ppm) =** 142.93, 129.12, 120.83, 117.61.

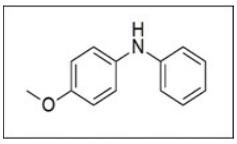

4-methoxy-N-phenylaniline [8] [9]
^{1}H NMR (500 MHz, $CDCl_3$) δ (ppm) = 7.18 (2H, dd, *J* = 8.4, 7.4 Hz), 7.06 (2H, d, *J* = 8.1 Hz), 6.87 (2H, d, *J* =7.9 Hz), 6.87 – 6.77 (3H, m), 5.49 (1H, s), 3.79 (3H, s). **^{13}C NMR (125 MHz, $CDCl_3$) δ (ppm) =** 155.21, 145.42, 135.77, 129.28, 122.13, 119.5, 115.7, 114.6, 55.7.

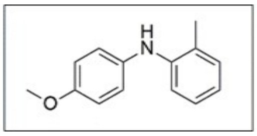

***N*-(4-methoxyphenyl)-2-methylaniline** [9]
^{1}H NMR (500 MHz, $CDCl_3$) δ (ppm) = 7.14 (1H, d, *J* = 7.6 Hz), 7.06 (1H, t, *J*= 8.7 Hz), 6.93 –6.89 (3H, m), 6.83 (2H, d, *J* = 8.0 Hz), 6.79 (1H, t, *J* = 8.4 Hz), 3.79 (3H, s), 2.24 (3H, s). **^{13}C NMR (125 MHz, $CDCl_3$) δ (ppm) =** 155.21, 143.42, 136.42, 130.91, 127.09, 125.53, 122.31, 120.23, 115.40, 114.91, 55.95, 18.21.

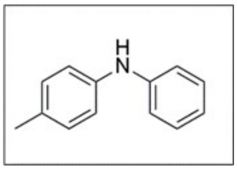

4-methyl-N-phenylaniline [9]
^{1}H NMR (500 MHz, $CDCl_3$) δ (ppm) = 7.20 (2H, t, *J* = 8.6 Hz), 7.05 (2H, d, *J* = 8.3 Hz), 7.01 – 6.94 (4H, m), 6.85 (1H, t, *J* = 7.3 Hz), 5.56 (1H, s), 2.29 (3H, s). **^{13}C NMR (125 MHz, $CDCl_3$) δ (ppm) =** 143.73, 140.15, 130.61, 129.17, 120.18, 118.74, 116.78, 20.79.

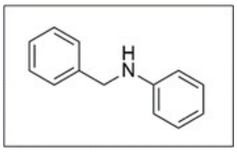

***N*-benzylaniline [10]**
^{1}H NMR (500 MHz, $CDCl_3$) δ (ppm) = 7.39 – 7.28 (4H, m), 7.26 (1H, dt, *J* = 6.3, 3.3 Hz), 7.17 (2H, tt, *J* =7.9, 1.9 Hz), 6.75 (1H, t, *J* = 7.3 Hz), 6.70 (2H, dd, *J* = 8.5, 0.9 Hz), 4.30 (2H, s). **^{13}C NMR (125 MHz, $CDCl_3$) δ (ppm) =** 147.94, 139.22, 129.15, 128.53, 127.34, 127.13, 117.41, 112.77, 48.43.

Figure S5: Sonogashira and Buchwald-Hartwig coupling substrate study products.

9. ^{1}H NMR and ^{13}C NMR Spectra

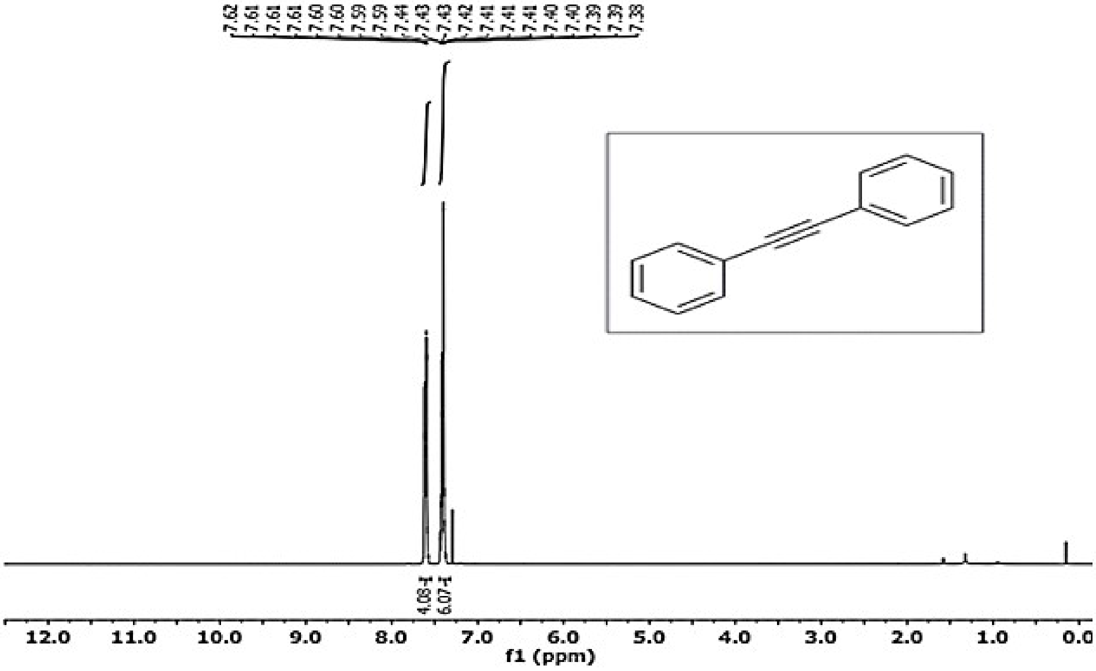

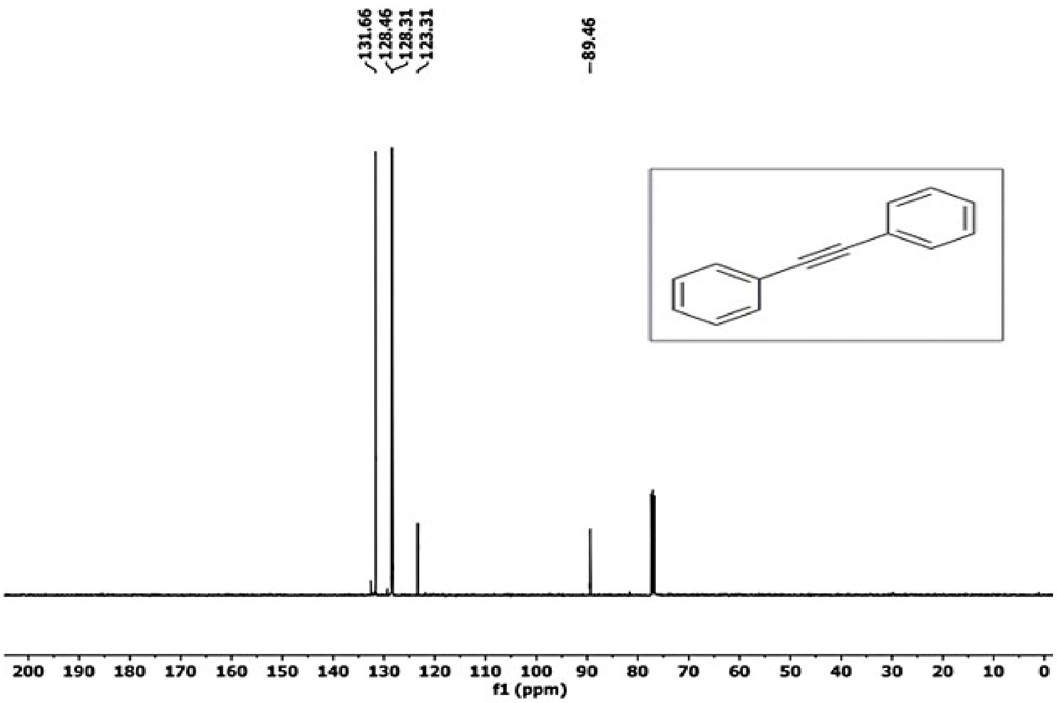
131.66
128.46
128.31
123.31
89.46
200 190 180 170 160 150 140 130 120 110 100 90 80 70 60 50 40 30 20 10 0
f1 (ppm)

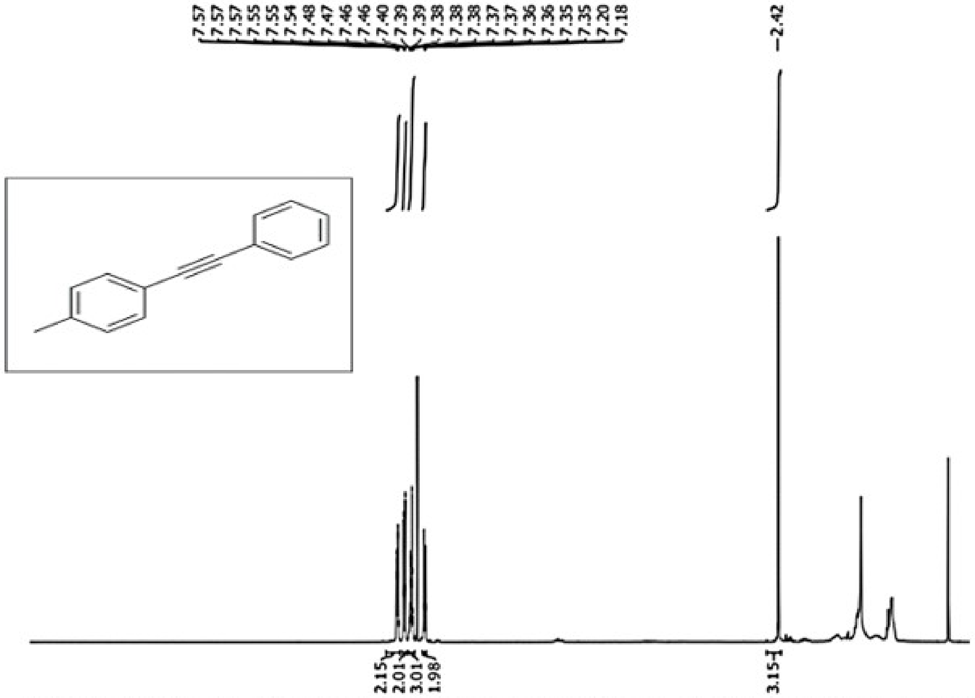
7.57
7.57
7.57
7.55
7.55
7.54
7.48
7.47
7.46
7.46
7.40
7.39
7.39
7.38
7.38
7.38
7.37
7.37
7.36
7.36
7.35
7.35
7.20
7.18
2.42
2.15
2.01
3.01
1.98
3.15
12.0 11.0 10.0 9.0 8.0 7.0 6.0 5.0 4.0 3.0 2.0 1.0 0.0
f1 (ppm)

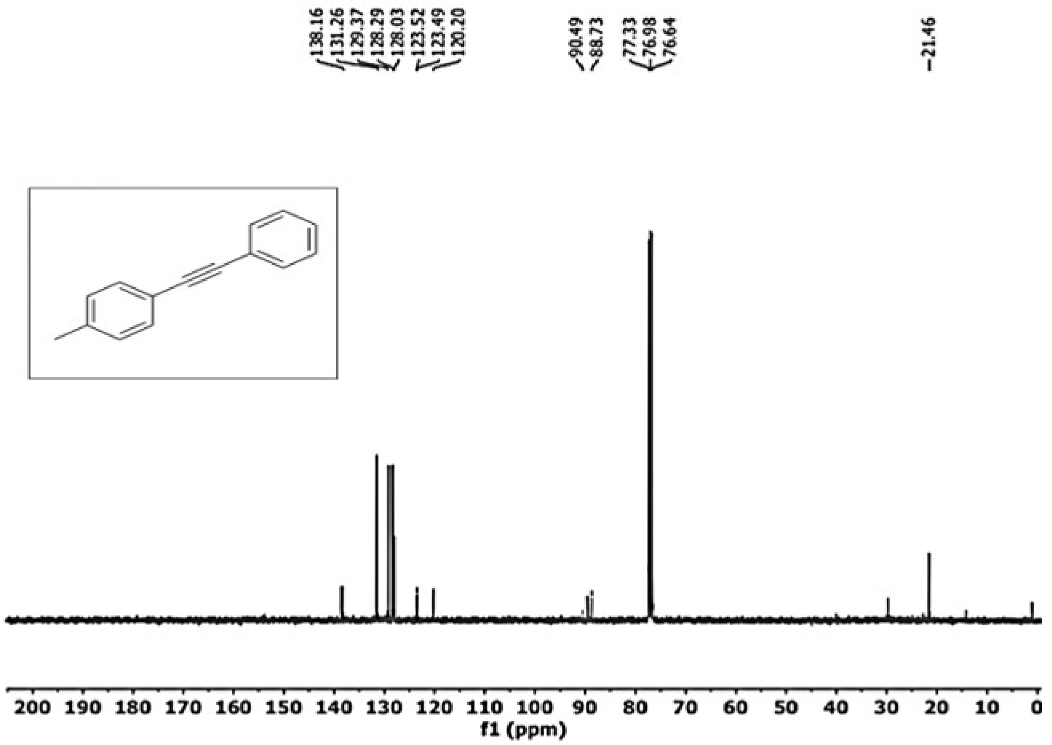
138.16
131.26
129.37
128.29
128.03
123.52
123.49
120.20
90.49
88.73
77.33
76.98
76.64
21.46
200 190 180 170 160 150 140 130 120 110 100 90 80 70 60 50 40 30 20 10 0
f1 (ppm)

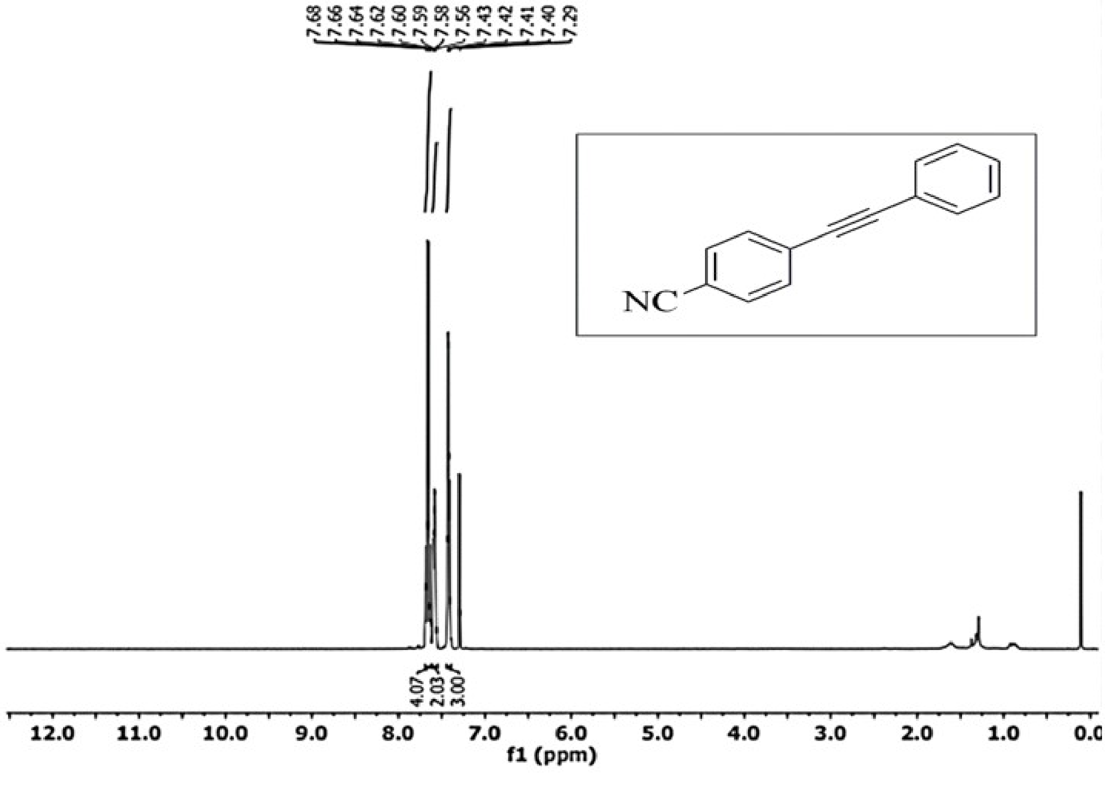
7.68
7.66
7.64
7.62
7.60
7.59
7.58
7.56
7.43
7.42
7.41
7.40
7.29
NC
4.07
2.03
3.00
12.0 11.0 10.0 9.0 8.0 7.0 6.0 5.0 4.0 3.0 2.0 1.0 0.0
f1 (ppm)

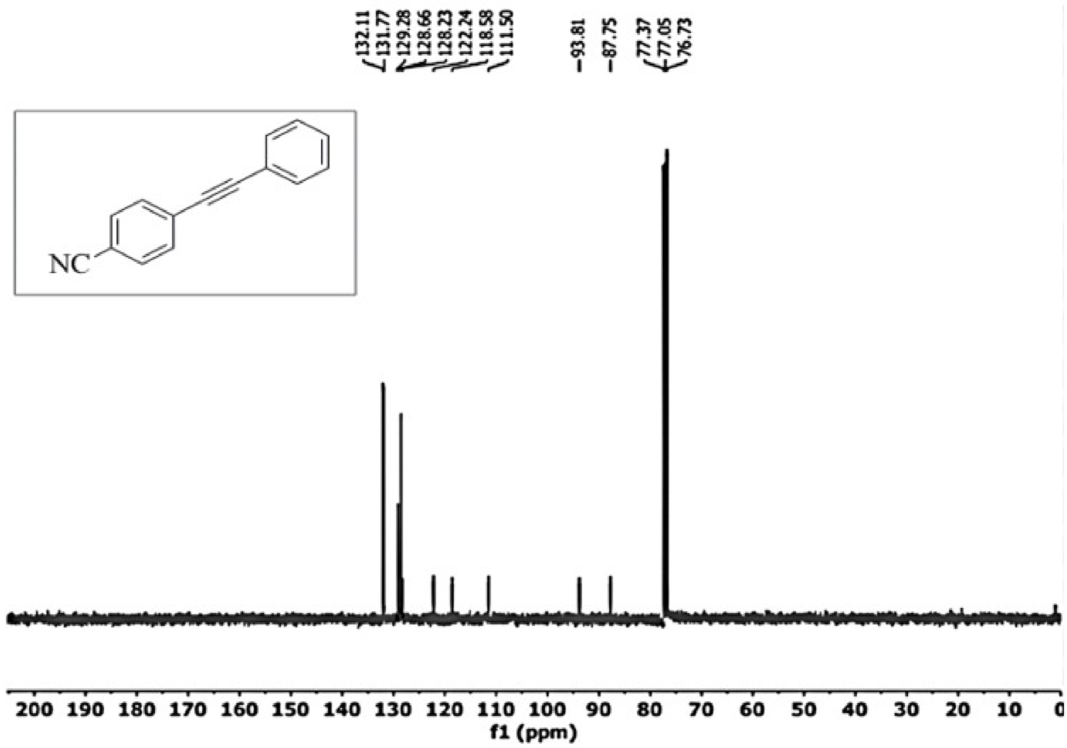
132.11
131.77
129.28
128.66
128.23
122.24
118.58
111.50
93.81
87.75
77.37
77.05
76.73
NC
200 190 180 170 160 150 140 130 120 110 100 90 80 70 60 50 40 30 20 10 0
f1 (ppm)

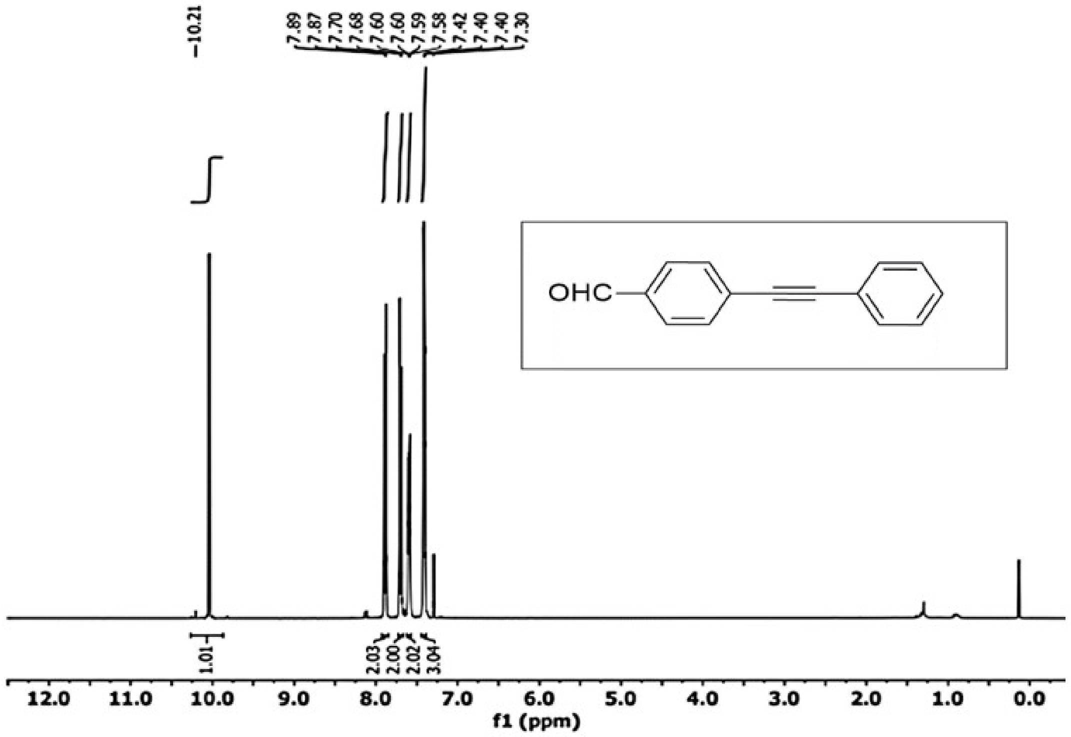
10.21
7.89
7.87
7.70
7.68
7.60
7.60
7.59
7.58
7.42
7.40
7.40
7.30
OHC
1.01
2.03
2.00
2.02
3.04
12.0 11.0 10.0 9.0 8.0 7.0 6.0 5.0 4.0 3.0 2.0 1.0 0.0
f1 (ppm)

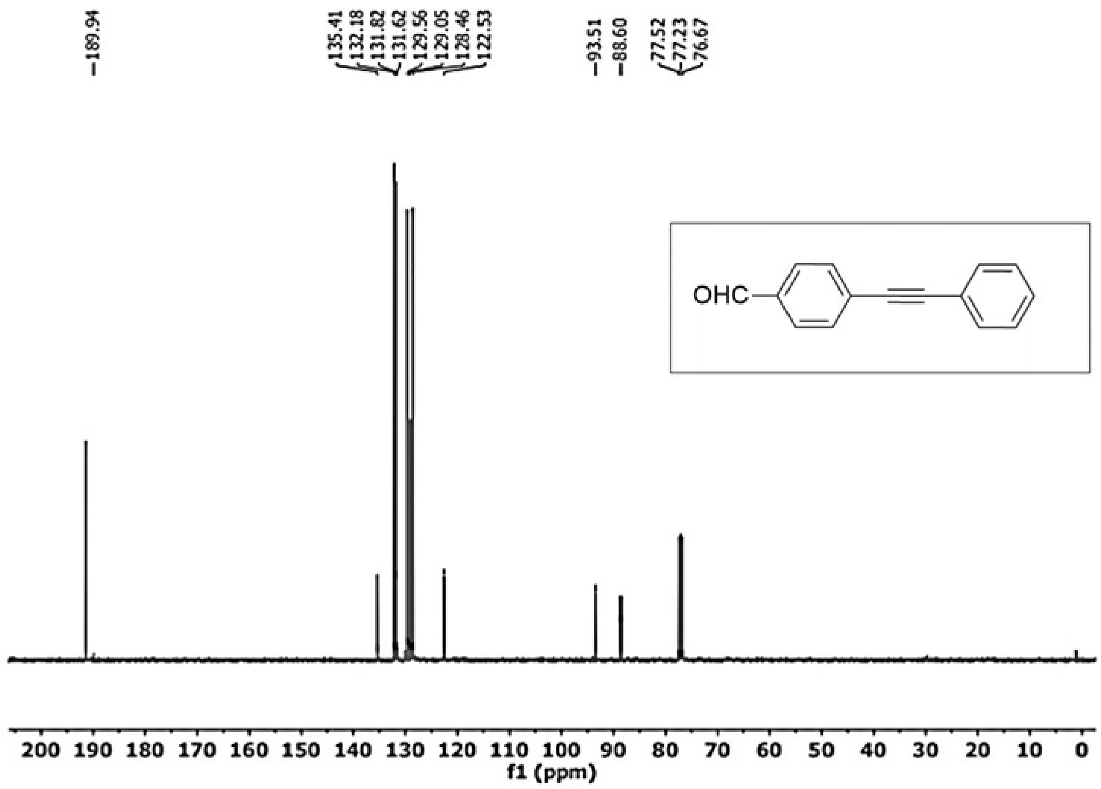
189.94
135.41
132.18
131.82
131.62
129.56
129.05
128.46
122.53
93.51
88.60
77.52
77.23
76.67
OHC
200 190 180 170 160 150 140 130 120 110 100 90 80 70 60 50 40 30 20 10 0
f1 (ppm)

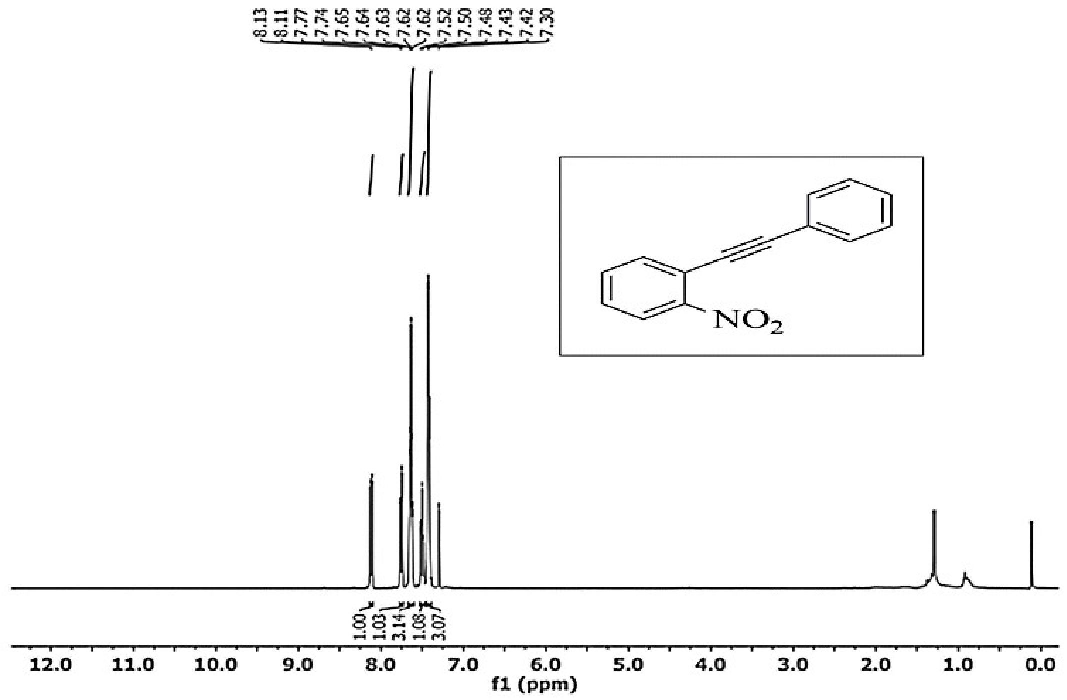
8.13
8.11
7.77
7.74
7.65
7.64
7.63
7.62
7.62
7.52
7.50
7.48
7.43
7.42
7.30
NO2
1.00
1.03
3.14
1.08
3.07
12.0 11.0 10.0 9.0 8.0 7.0 6.0 5.0 4.0 3.0 2.0 1.0 0.0
f1 (ppm)

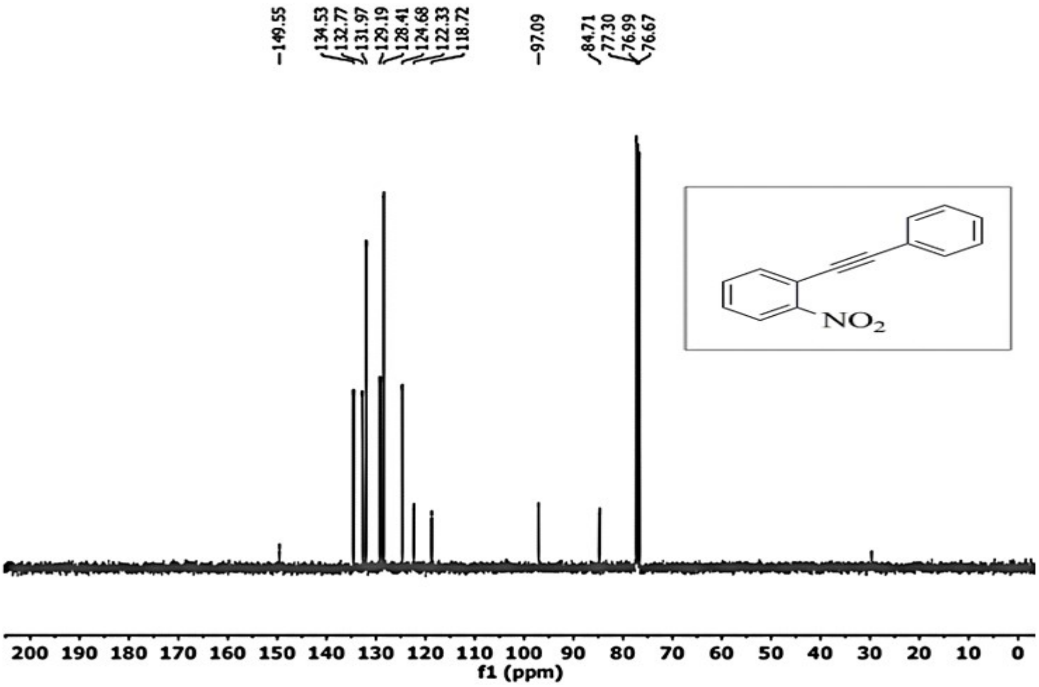
149.55
134.53
132.77
131.97
129.19
128.41
124.68
122.33
118.72
97.09
84.71
77.30
76.99
76.67
NO_2
200 190 180 170 160 150 140 130 120 110 100 90 80 70 60 50 40 30 20 10 0
f1 (ppm)

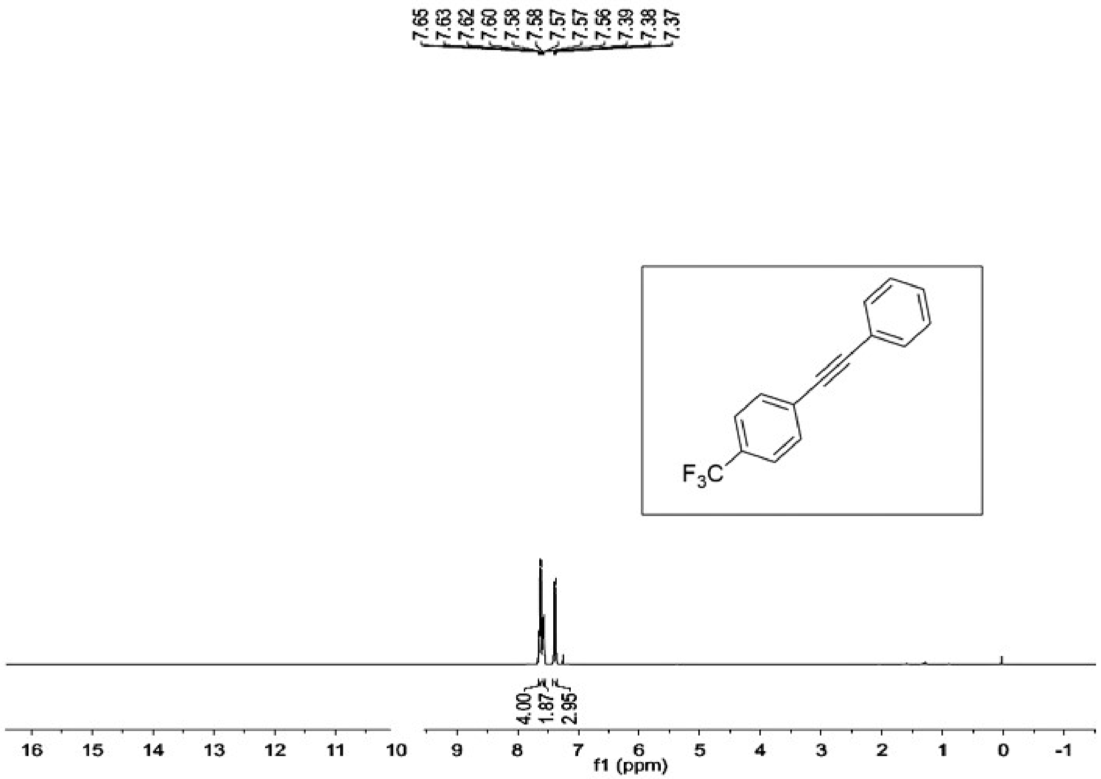
7.65
7.63
7.62
7.60
7.58
7.58
7.57
7.57
7.56
7.39
7.38
7.37
F_3C
4.00
1.87
2.95
16 15 14 13 12 11 10 9 8 7 6 5 4 3 2 1 0 -1
f1 (ppm)

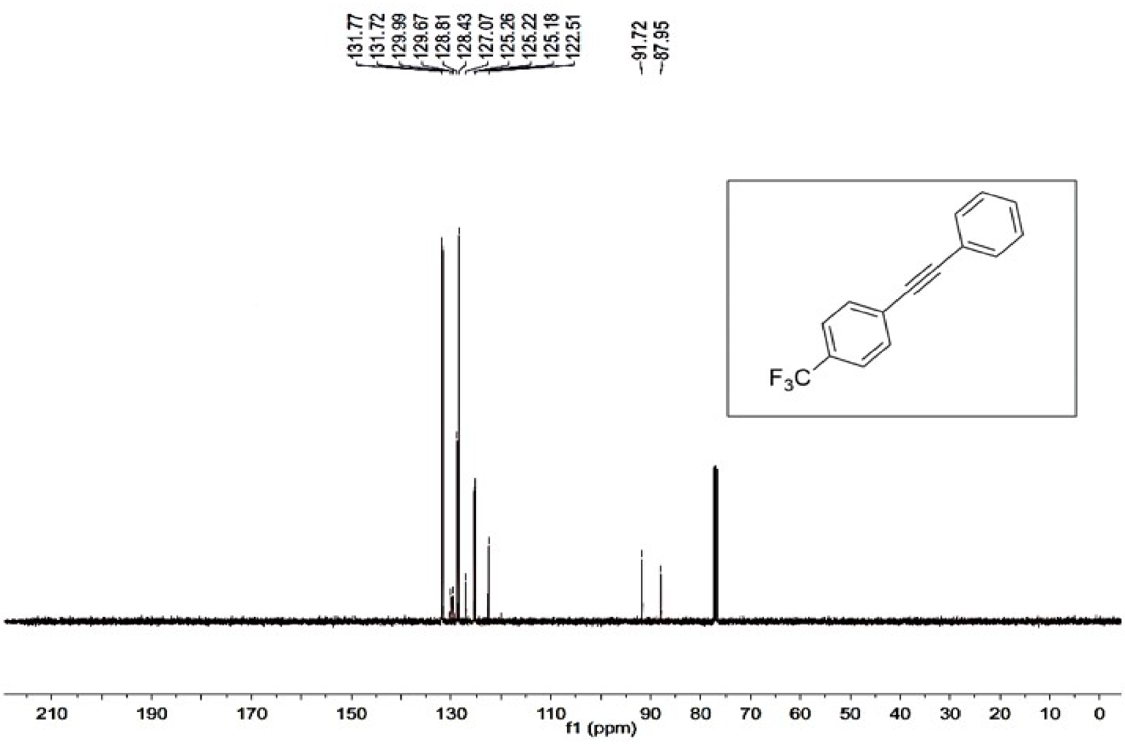
131.77
131.72
129.99
129.67
128.81
128.43
127.07
125.26
125.22
125.18
122.51
91.72
87.95
F3C
210
190
170
150
130
110
90
80
70
60
50
40
30
20
10
0
f1 (ppm)

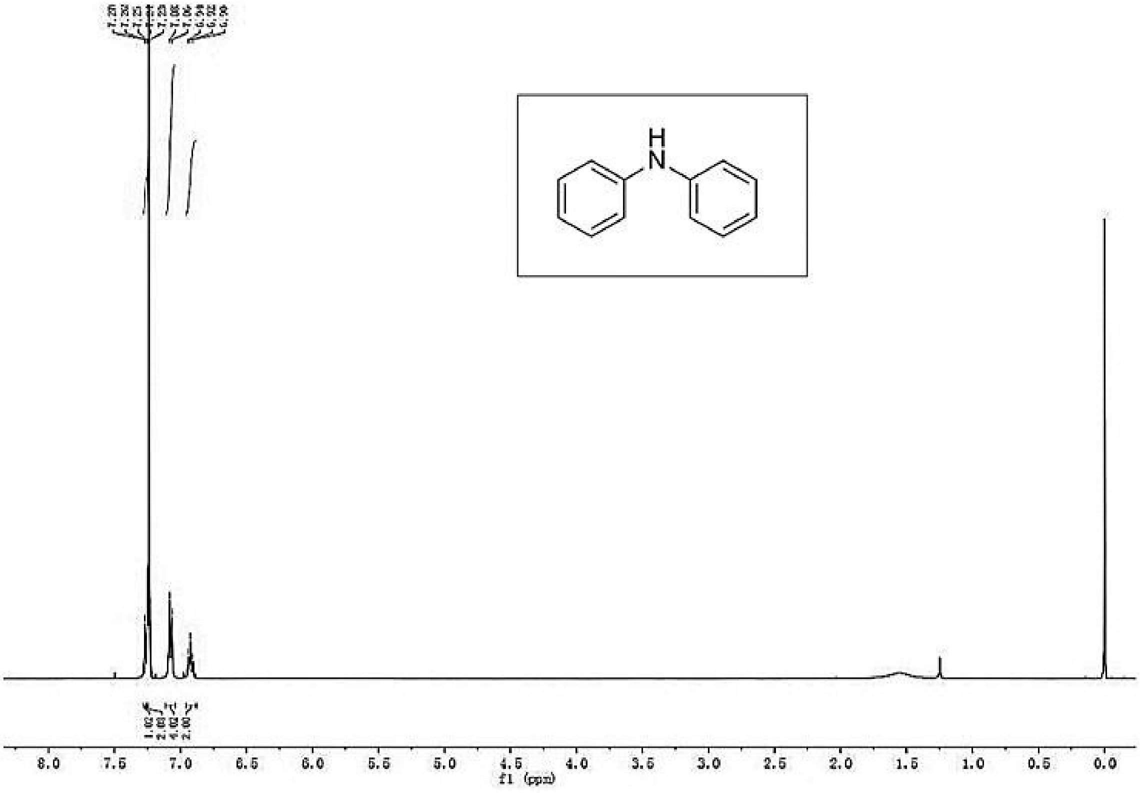

8.0 7.5 7.0 6.5 6.0 5.5 5.0 4.5 4.0 3.5 3.0 2.5 2.0 1.5 1.0 0.5 0.0
f1 (ppm)

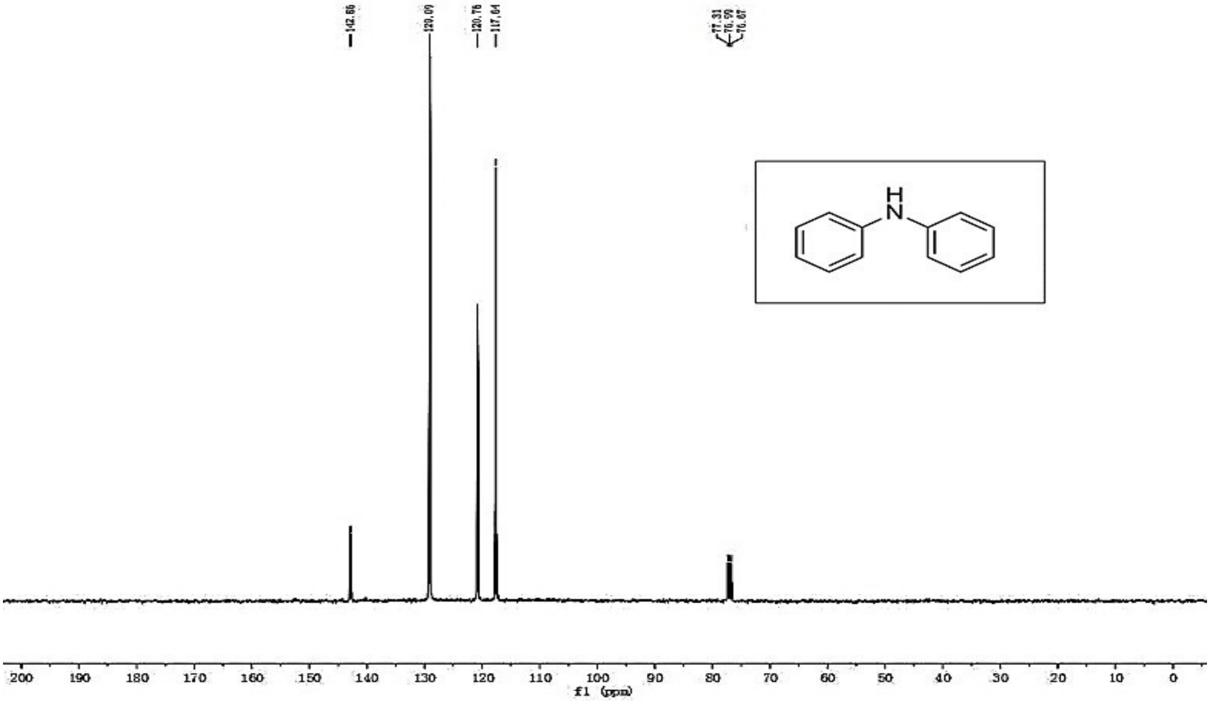

200 190 180 170 160 150 140 130 120 110 100 90 80 70 60 50 40 30 20 10 0
f1 (ppm)

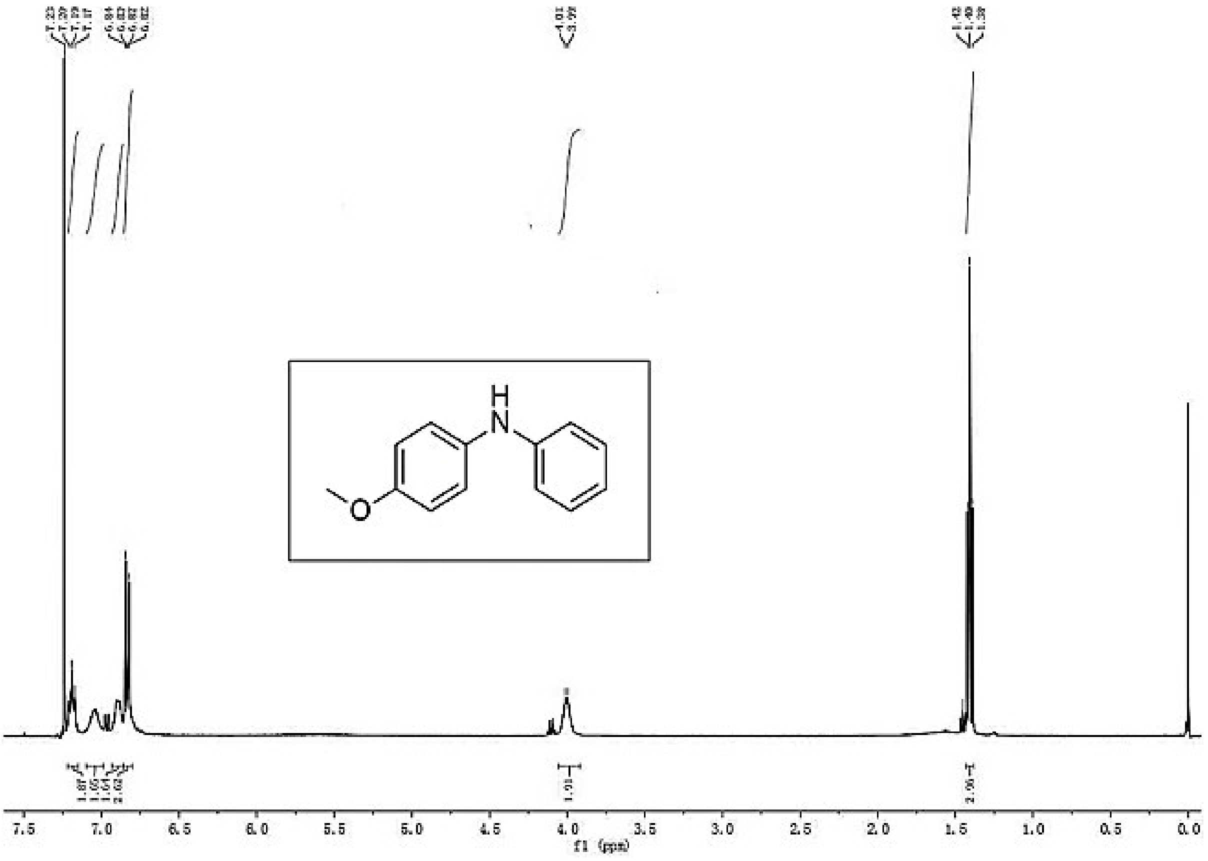
H
N
O
f1 (ppm)

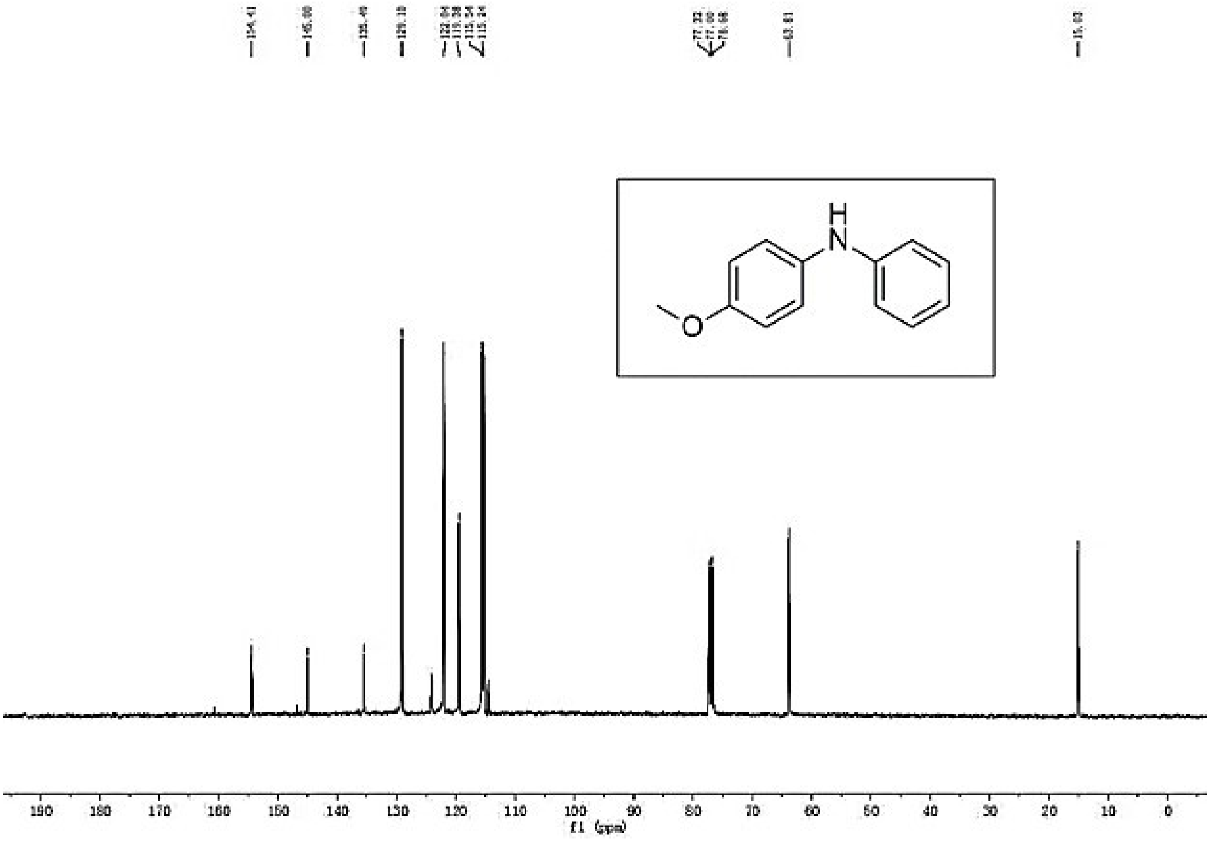
H
N
O
f1 (ppm)

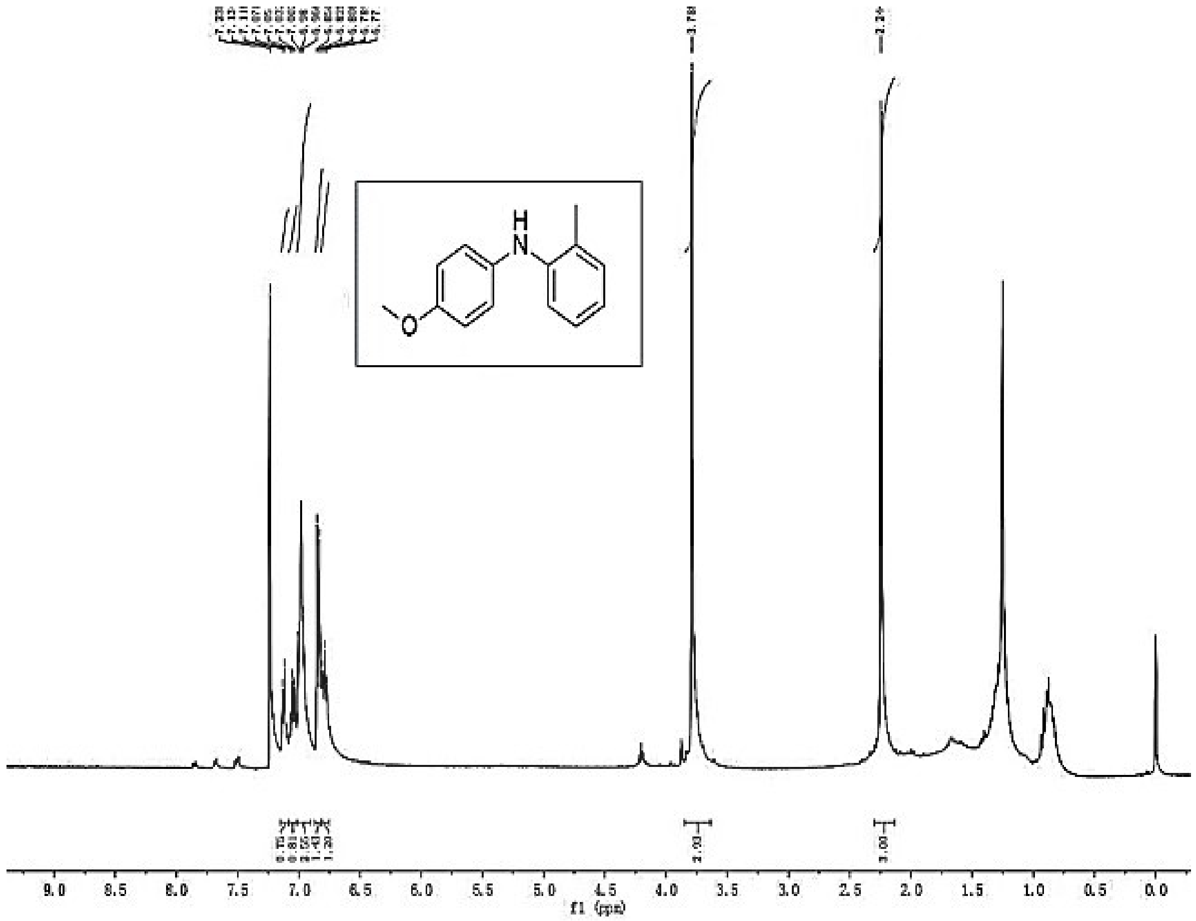

H
O
9.0 8.5 8.0 7.5 7.0 6.5 6.0 5.5 5.0 4.5 4.0 3.5 3.0 2.5 2.0 1.5 1.0 0.5 0.0
f1 (ppm)

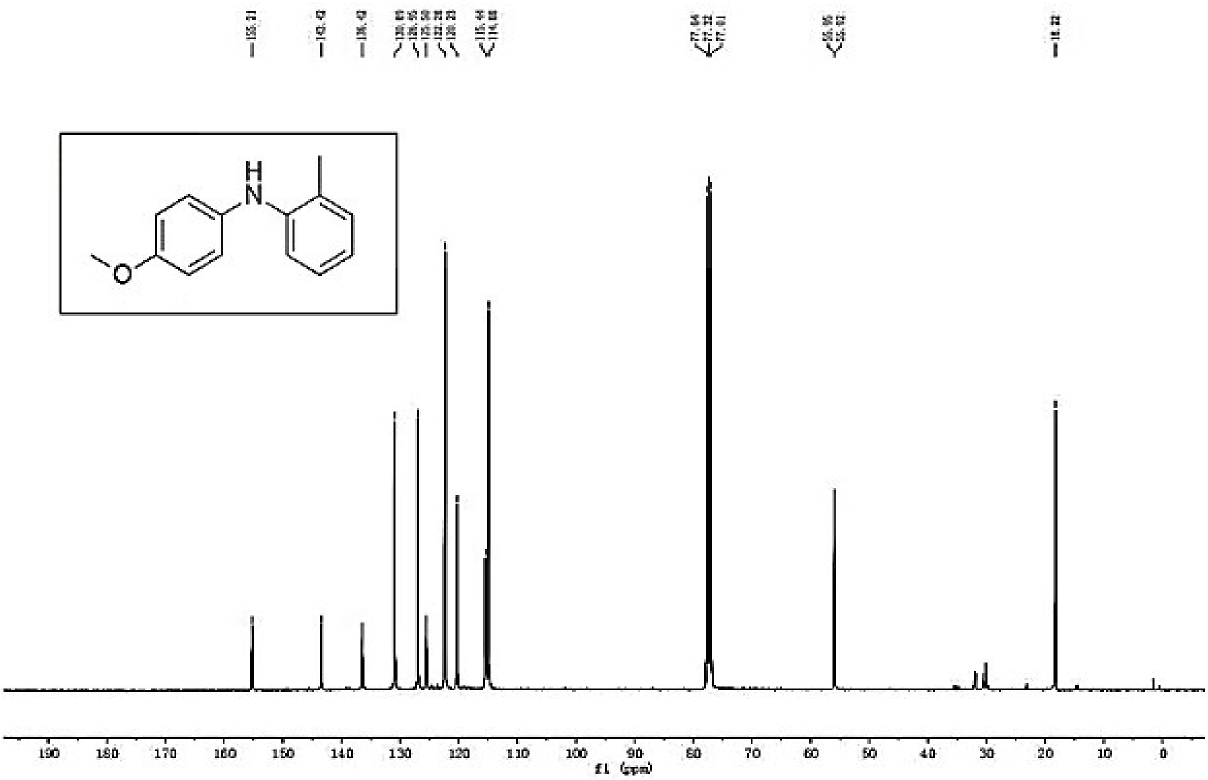

H
O
190 180 170 160 150 140 130 120 110 100 90 80 70 60 50 40 30 20 10 0
f1 (ppm)

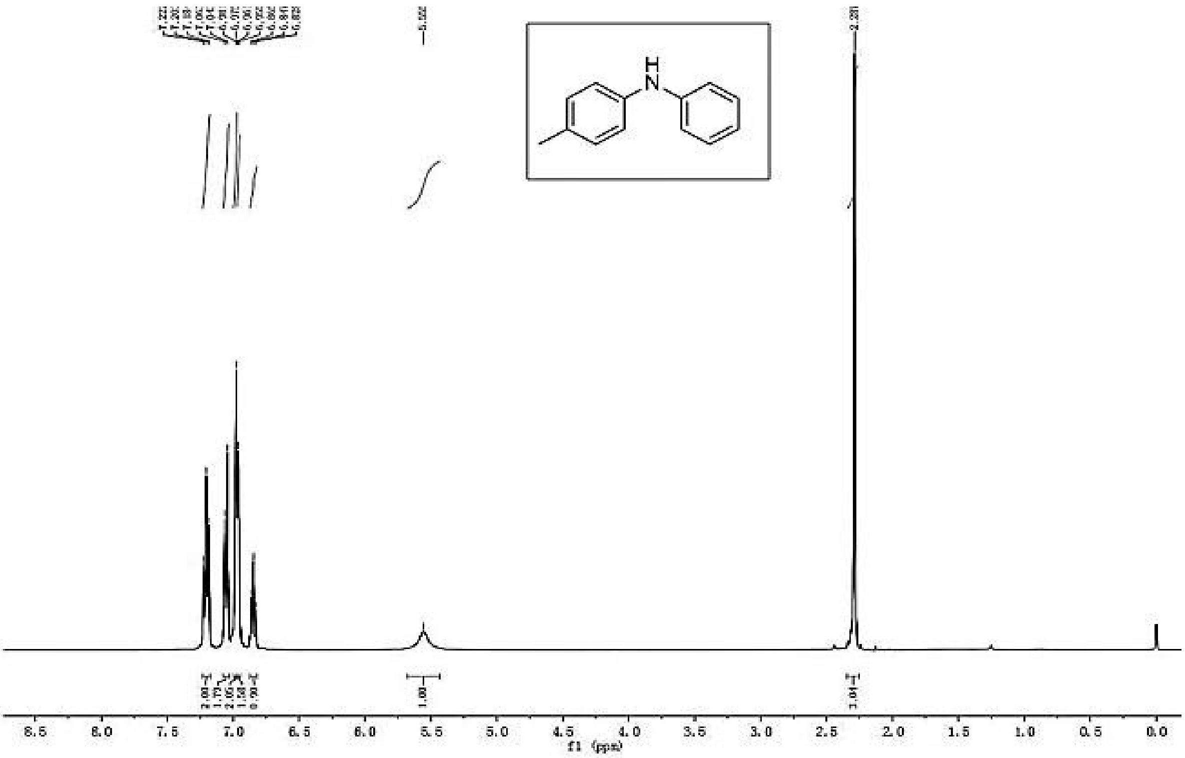
f1 (ppm)

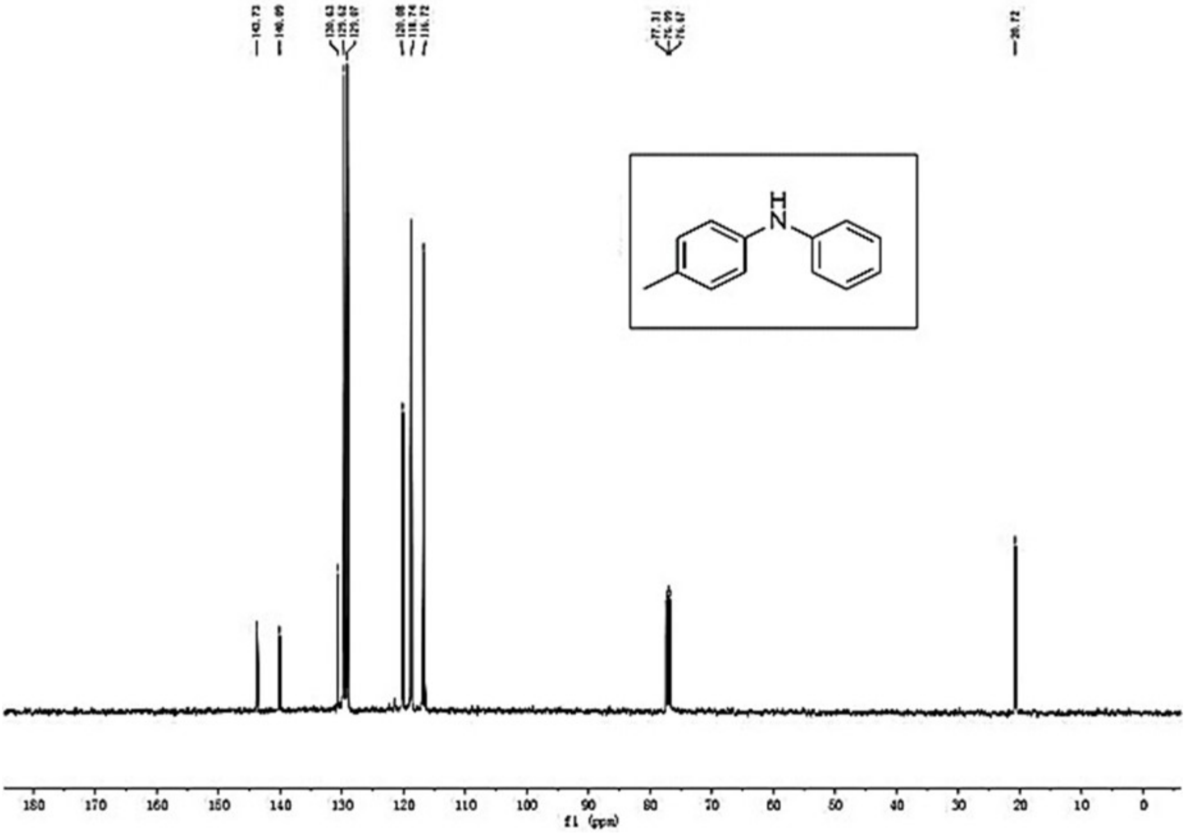
f1 (ppm)

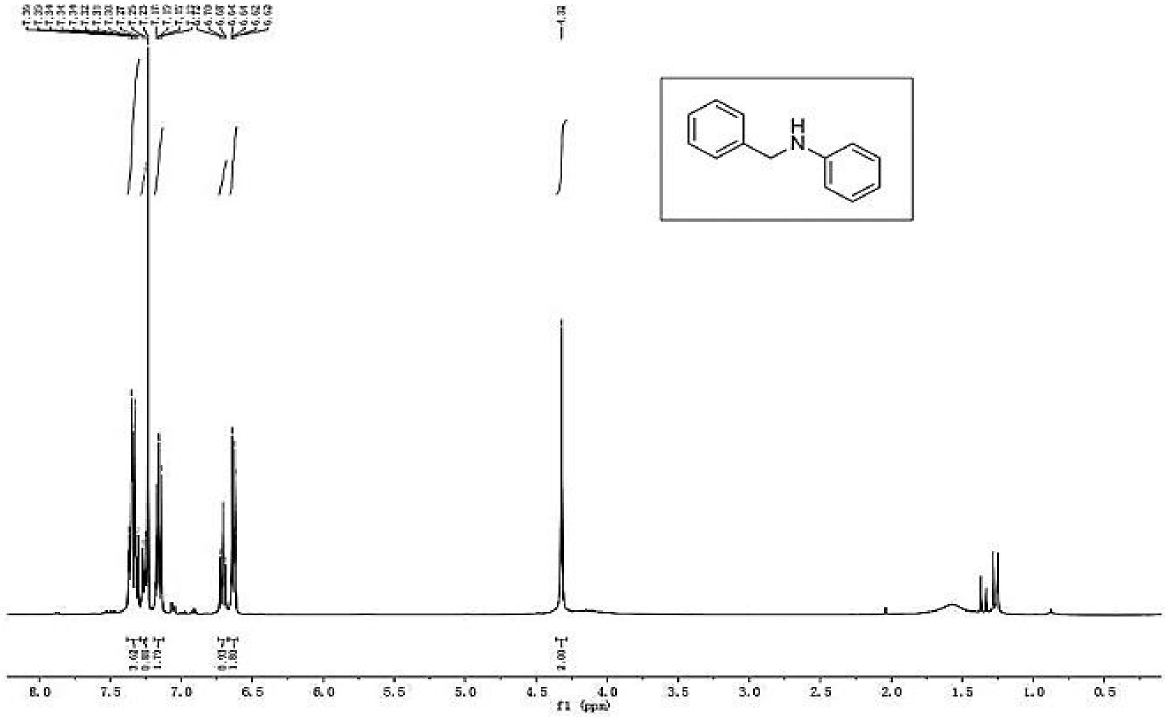

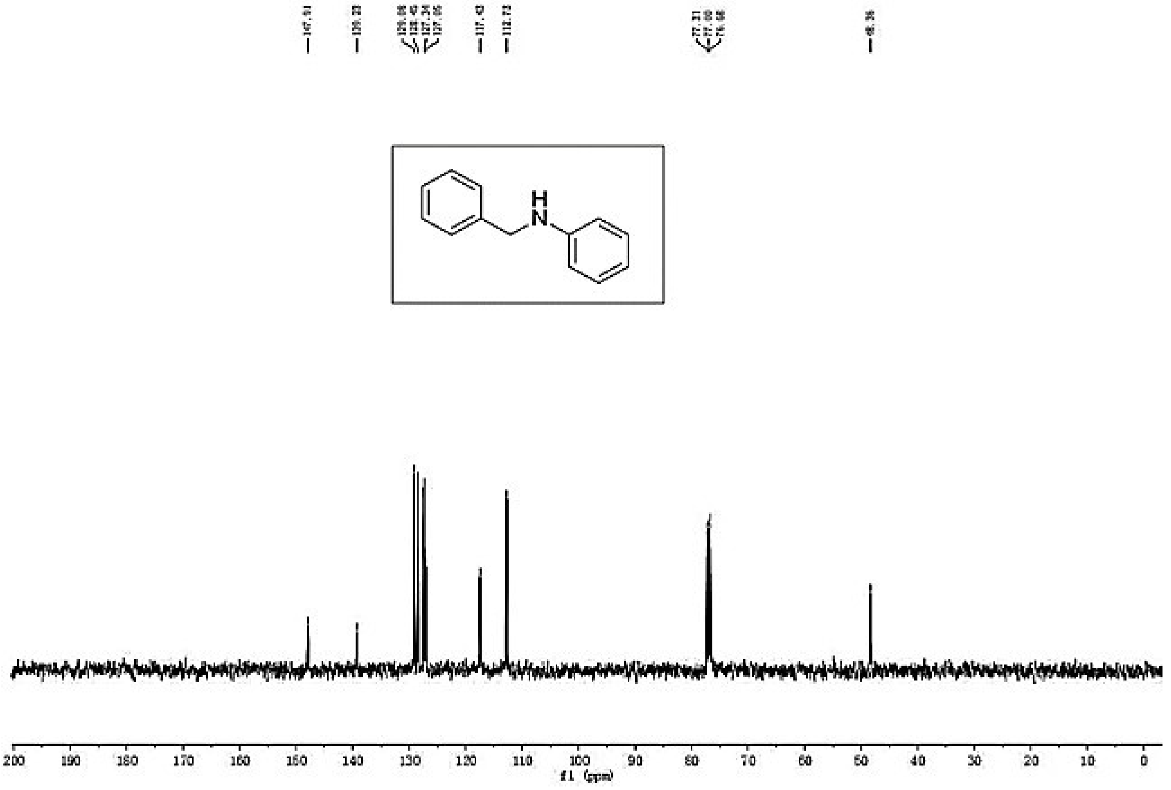

Figure S6: NMR spectra of the substrate study products of Sonogashira and Buchwald-Hartwig coupling.
